# Ontogenetic development of intestinal length and relationships to diet in an Australasian fish family (Terapontidae)

**DOI:** 10.1186/1471-2148-13-53

**Published:** 2013-02-25

**Authors:** Aaron M Davis, Peter J Unmack, Bradley J Pusey, Richard G Pearson, David L Morgan

**Affiliations:** 1 Centre for Tropical Water and Aquatic Ecosystem Research (TropWATER), Townsville, QLD, 4811, Australia; 2 National Evolutionary Synthesis Center, Durham, NC, 27705-4667, USA; 3 Centre of Excellence in Natural Resource Management, University of Western Australia, Albany, 6330, Australia; 4 School of Marine and Tropical Biology, James Cook University, Townsville, QLD, 4811, Australia; 5 Freshwater Fish Group and Fish Health Unit, Murdoch University, South St., Murdoch, WA, 6150, Australia

**Keywords:** Dietary radiation, Allometry, Morphological evolution, Phylogenetic comparative method, Herbivory-detritivory

## Abstract

**Background:**

One of the most widely accepted ecomorphological relationships in vertebrates is the negative correlation between intestinal length and proportion of animal prey in diet. While many fish groups exhibit this general pattern, other clades demonstrate minimal, and in some cases contrasting, associations between diet and intestinal length. Moreover, this relationship and its evolutionary derivation have received little attention from a phylogenetic perspective. This study documents the phylogenetic development of intestinal length variability, and resultant correlation with dietary habits, within a molecular phylogeny of 28 species of terapontid fishes. The Terapontidae (grunters), an ancestrally euryhaline-marine group, is the most trophically diverse of Australia’s freshwater fish families, with widespread shifts away from animal-prey-dominated diets occurring since their invasion of fresh waters.

**Results:**

Description of ontogenetic development of intestinal complexity of terapontid fishes, in combination with ancestral character state reconstruction, demonstrated that complex intestinal looping (convolution) has evolved independently on multiple occasions within the family. This modification of ontogenetic development drives much of the associated interspecific variability in intestinal length evident in terapontids. Phylogenetically informed comparative analyses (phylogenetic independent contrasts) showed that the interspecific differences in intestinal length resulting from these ontogenetic developmental mechanisms explained ~65% of the variability in the proportion of animal material in terapontid diets.

**Conclusions:**

The ontogenetic development of intestinal complexity appears to represent an important functional innovation underlying the extensive trophic differentiation seen in Australia’s freshwater terapontids, specifically facilitating the pronounced shifts away from carnivorous (including invertebrates and vertebrates) diets evident across the family. The capacity to modify intestinal morphology and physiology may also be an important facilitator of trophic diversification during other phyletic radiations.

## Background

Morphological divergence associated with dietary shifts has played a major role in the phyletic radiation of many vertebrates [1-5]. These evolutionary changes in diet and trophic morphology can occur rapidly [6,7], even within ecological timescales [8]. However, the frequency with which particular dietary modes have evolved varies considerably across different vertebrate lineages. While plant-based diets have a broad taxonomic distribution among mammals (>25%) [9], the occurrence of herbivory is much more restricted (2–5% of species) amongst other vertebrate groups [6,10]. Despite the wide array of feeding modes amongst fishes and the biomass dominance of herbivorous and detritivorous fishes in many assemblages [11,12], the development of these non-animal prey based trophic habits has been an infrequent evolutionary phenomenon, largely confined to a few families of teleosts [10,13-16]. The morphological and physiological specializations that facilitate these trophic shifts have accordingly attracted considerable interest from ecologists and evolutionary biologists [10,16-19].

One of the most widely identified ecomorphological relationships between vertebrate morphology and ecology, and one particularly relevant to dietary radiations involving shifts from carnivory to plant-detrital diets, is intestinal length. The vertebrate digestive tract represents a functional link between foraging (energy intake) and energy management and allocation, but is energetically costly to maintain, and may account for 20–25% of an animal’s metabolic rate [20]. A core prediction of digestive theory [sensu [18,21] is that the consumption of food with a high content of indigestible material results in an increase in gut dimensions. Numerous studies have shown that digestive tracts across all vertebrate classes tend to be shortest in carnivores, intermediate in omnivores and longest in herbivores and detritivorous species, [20,22,23]. The functional significance of this association lies in the need for species on diets that are low in protein and high in roughage to have longer guts in order to ingest large volumes of low-quality food, increase absorptive surface area and maximise digestive efficiency [19]. While a range of fish families display this diet-morphology relationship [17,24-29], other fish groups demonstrate minimal, and in some cases contrasting relationships between intestinal length and diet [30,31].

Much of the literature on diet-intestinal length relationships makes little acknowledgment of the evolutionary history of the studied species [18]. Species sharing a common ancestor are not evolutionarily independent, and phylogenetic proximity voids the assumption of sample independence underpinning many conventional statistical tests, thereby creating difficulties in attributing morphological-ecological relationships to adaptive causes rather than phylogenetic artefacts [32]. Applying caution to inferences drawn from phylogenetically naive diet-intestinal length studies is being increasingly advocated [18,24,25,33]. While an abundance of comparative ecomorphological studies of oral kinematics, food procurement and dietary habits in vertebrates has recently emerged [34-37], the association between diet and intestinal length has received surprisingly little phylogenetically informed attention; although recent exceptions have occurred [25,26].

While developmental plasticity has long been posited as a driver of the origin and diversification of novel traits [38], study of the evolutionary and developmental processes underpinning interspecific differences in intestinal length has been largely neglected. Interspecific variations in intestinal length between closely related species are largely driven by variations in allometric intestinal growth during ontogeny [39,40]. Substantive allometric increases in intestinal length typically involve additional intestinal looping or convolution that must be accommodated in the body cavity [41]. Previous research has suggested looping patterns are not random, with an underlying phylogenetic component, so that patterns of development of intestinal looping have been used to reconstruct the phylogenetic systematics of a number of fish lineages [41-43]. Yamaoka [43] noted that use of intestinal complexity as a tool in systematic research involves a ‘two-storey’ structure, with the first storey comprising a qualitative aspect (coiling pattern), and the second storey composed of the quantitative (functional) component of intestinal length. To our knowledge, integration of ontogenetic development patterns with molecular phylogenetic reconstruction and comparative approaches has not been attempted. Concurrent appraisal of the ontogenetic processes producing variation in intestinal length, and the functional significance of these processes (i.e., associations with diet) in a phylogenetic context is similarly lacking.

**Figure 1:**
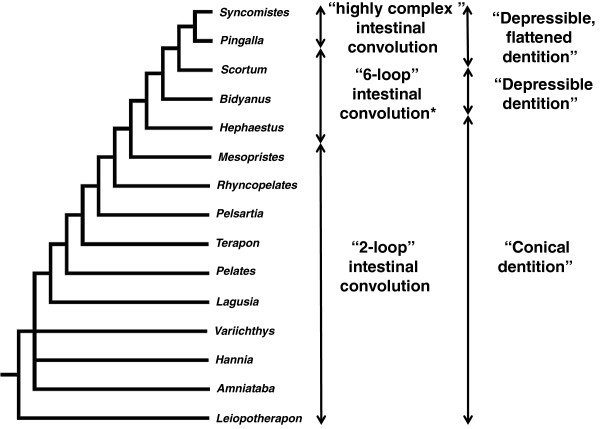
**Cladogram depicting terapontid generic relationships derived from comparative morphology, adapted from Vari **[42]**.** Cladogram depicting terapontid generic relationships derived from comparative morphology, adapted from [42], showing intestinal convolution and dentition characters used to differentiate genera. Note that *Amniataba, Hannia* and *Variichthys* form an unresolved trichotomy. Vari [42] also identified two distinct sub-clades within the genus *Hephaestus* – “genus a” which develops a “6-loop” intestinal pattern, and “genus b” which retains the plesiomorphic “2-loop” intestine.

Northern Australia’s Terapontidae (grunters) offer a promising candidate for examining the relationship between intestinal length and diet, and the phylogenetic context for ontogenetic development of intestinal length. The Terapontidae is one of the most speciose and trophically diverse of Australia’s freshwater fish families, exhibiting carnivorous, omnivorous, herbivorous and detritivorous feeding habits [44]. A genus-level phylogeny of the family by Vari [45] relied heavily on differences in ontogenetic development of intestinal configuration as a diagnostic character. Vari’s morphological character analysis suggested a sequence of four intestinal patterns of increasing complexity, beginning at the plesiomorphic condition of a simple two-loop intestine throughout life history in the genera *Leiopotherapon*, *Amniataba*, *Hannia*, *Variichthys*, *Lagusia*, *Terapon*, *Pelates*, *Pelsartia*, *Rhyncopelates* and *Mesopristes* (Figure 1). The genera *Hephaestus*, *Bidyanus* and *Scortum* have an intermediate “six-loop” pattern. Juveniles of these genera initially possess the “two-loop” pattern before undergoing an ontogenetic elongation and folding to produce more complex patterns as adults. Vari noted that this pattern appears to have been secondarily lost in a subunit of *Hephaestus* referred to as *Hephaestus* “genus b”. The adult life stages of the genera *Pingalla* and *Syncomistes* purportedly undergo a further ontogenetic shift to produce a highly convoluted and elaborate intestinal pattern, with the final and most complex intestinal pattern unique (autoapomorphic) to *Syncomistes*. Juveniles of the species in *Pingalla* and *Syncomistes* possess a similar intestinal convolution to adults of genera exhibiting the adult “six-loop” pattern, with Vari presuming these species pass through the simple “two-loop” pattern earlier in ontogeny.

A recent molecular-based phylogeny suggests a different topology for this phylogeny, as well as substantial lineage and dietary diversification, particularly the adoption of plant and detritus-based diets, upon a single historical invasion of fresh water environments by euryhaline-marine terapontids [45]. Here we utilise a suite of phylogenetic comparative methods to address two study aims: firstly we re-examine the process of ontogenetic development of intestinal length in Terapontidae within the context of a molecular phylogeny. Patterns of ontogenetic intestinal configuration are described and then combined with ancestral character state reconstruction to examine the evolutionary history of intestinal complexity within terapontids, including the number of gains/losses of particular intestinal patterns within the family. Secondly, in line with predictions of digestive theory, we predict shorter intestinal length in species that consume higher proportions of animal prey than those consuming greater amounts of plant and/or detrital material. If this hypothesized relationship exists, it will provide evidence for dietary ecomorphological diversification, based on modification of intestinal length, which is likely to be a significant driver of the phyletic and trophic radiation evident in Australia’s freshwater terapontids.

## Methods

### Taxon sampling, molecular markers and phylogeny reconstruction

A phylogenetic analysis of 28 terapontid species was performed based on nuclear and mitochondrial DNA (mtDNA) sequences from Davis and others [45]. The ingroup consisted of 28 species, including nine Australian marine-euryhaline species, all genera and 18 of 24 species of Australian freshwater terapontids, and one species present only in New Guinea. Two representative sequences of one species (*Hannia greenwayi*) were included due to their different placement in the topology. Distribution of species used in this study in relation to Davis and others [45] are presented in supporting information (see Additional file 1: Figure S1). On the basis of earlier stomach-content based classifications of diet, these selected species exhibit all of the major trophic habits displayed by Australia’s freshwater, euryhaline and marine terapontids: invertivory, generalised carnivory, omnivory, herbivory and detritivory-algivory [45].

Sequence data consisted of an 1141 base-pair (bp) fragment of the mtDNA gene cytochrome *b* (cyt*b*) and a 3896 and 905 bp fragment of the nuclear recombination activation genes RAG1 and RAG2 (hereafter referred to as RAG) respectively for a total of 5942 bp for each individual included in our study. We used our previous dataset [45]; Dryad Digital Repository doi:http://10.5061/dryad.4r7b7hg1, trimmed out taxa for which we lacked ontogenetic data and realigned the dataset. Cyt*b* was aligned by eye while RAG sequences were aligned using the online version of MAFFT 6.822 [46] using the accurate G-INS-i algorithm with the scoring matrix for nucleotide sequences set to 1PAM/K=2, a gap opening penalty of 1.53 and an offset value of 0.1. Combined partitioned phylogenetic analyses were performed with maximum likelihood (ML) using GARLI 2.0 [47]. We identified the best-fitting model of molecular evolution using the Akaike Information Criterion (AIC) in Modeltest 3.7 [48] using PAUP* 4.0b10 [49]. For cyt*b* Modeltest identified TrN+I+G as the best model and for RAG GTR+I+G was the best model. We ran GARLI with 10 search replicates using the default settings with two partitions representing cyt*b* and RAG with their respective models. For bootstrapping we ran 1000 replicates with the previous settings except that the options genthreshfortopoterm was reduced to 10,000 and treerejectionthreshold was reduced to 20 as suggested in the GARLI manual to speed up bootstrapping. The concatenated sequence data file and tree files were deposited in Dryad, doi:http://10.5061/dryad.h30t5. Trees were rooted with representatives from several related families based on the work of Yagishita and others [45,50].

### Specimen collection

Fish for dietary and morphometric quantification were collected from a number of fish survey studies conducted across fresh water and marine habitats across Australia [44] and Papua New Guinea, as well as being sourced from museum collections. Fish were preserved in either buffered formalin or ethanol. Larger specimens had incisions in the body wall or fixative injected via hypodermic syringe into the body cavity to aid fixation of internal organs.

### Intestinal coiling pattern description and intestinal length measurement

After weighing fish and measuring standard length (SL, in mm), specimens were dissected and the entire digestive system and viscera were removed from the body cavity. All terapontids possess a Y-shaped stomach with a straight descending limb from the oesophagus, followed by a blind sac at the bend of the stomach, which leads anteriorly to the pyloric limb on the left side of the body [42]. Intestinal convolution patterns posterior to the pyloric outlet were observed using a dissecting microscope and sketched and photographed from dorsal, ventral, left and right aspects. While Vari [42] described intestinal patterns from the left side of the body, we followed Yamaoka [43] by defining intestinal patterns from the ventral aspect, which facilitates definition of the bilaterally symmetrical body structure of fishes. After description of intestinal coiling structure, the intestine was carefully uncoiled to avoid stretching and intestinal length (IL) was measured as the distance from the pyloric outlet to the rectum. Species’ means for standard length and intestinal length were log_10_ transformed to homogenise variance prior to analysis and to increase data independence.

### Reconstructing the evolutionary history of terapontid intestinal length development

The historical patterns of terapontid intestinal development were hypothesized utilising ancestral character reconstruction techniques in Mesquite 2.75 [51]. We used the “Trace Over Trees” function in Mesquite, which reconstructs ancestral history on multiple phylogenies, to incorporate phylogenetic uncertainty in ancestral reconstructions of character states. In order to generate a collection of trees we used the Bayesian method BEAST 1.7.1 [52] and generated input files using BEAUti 1.7.1. The analysis used an uncorrelated lognormal relaxed molecular clock with rate variation following a tree prior using the speciation birth-death process, and the same models of sequence evolution for the nuclear and mtDNA partitions as per our ML analysis above. BEAST analyses were run for 50 million generations, with parameters logged every 100,000 generations. Multiple runs were conducted to check for stationarity and that independent runs were converging on a similar result. The treefile was summarized using TreeAnnotator 1.7.1 with the mean values placed on the maximum clade credibility tree. The first 10% of trees were removed as burn-in, providing 450 trees for reconstructing ancestral states, with ancestral states summarized onto the maximum clade credibility tree. States were summarized for each node by counting all trees with uniquely best states. If no state was more parsimonious than the other, the reconstruction at that node was classed as equivocal. The frequency of each state was reported for all trees containing that ancestral node, with the variability of inferred states among trees providing a measure of the degree to which ancestral state reconstructions for the node concerned are affected by uncertainty in tree topology and branch lengths. Adult intestinal configurations were coded as discrete (categorical) character states and optimised onto the molecular phylogeny. Because alternative methods of character state reconstruction can produce conflicting results, both maximum parsimony (MP) and maximum likelihood ML methods of ancestral state reconstruction were employed [53,54]. Parsimony ancestral state reconstruction, which minimizes the amount of character change given a tree topology and character state distribution, has been widely utilised but may over-represent confidence in ancestral character states [53]. For the MP analysis, character transitions were considered to be unordered (changes between any character state are equally costly). A character was assigned to a node if it created fewer steps, otherwise the node was considered equivocal.

ML ancestral character state reconstruction finds the ancestral states that maximize the probability that the observed states would evolve under a stochastic model of evolution [53,54]. A symmetrical Mk1 model [55], which assumes equal forward and backward character transition rates (i.e., all changes equally probable), was used as the evolutionary model. A major advantage of ML is that the analysis takes branch lengths into account, allows the uncertainty associated with each reconstructed ancestral state to be quantified, and is preferable for medium-sized trees [54,56]. Likelihood ratios at internal nodes were compared by pairs, and were reported as proportional likelihoods. While likelihoods do not necessarily translate into levels of statistical significance, a difference of 2 log units for a character (i.e., ~7.4 times more probable than any other alternative state) was employed to assign states at a node, otherwise the node was considered equivocal (defined as ‘the rule of thumb’) [54].

### Dietary data

Pronounced ontogenetic diet shifts in association with significant allometric growth in many diet-ecomorphological characters are a prominent feature of terapontid ontogenetic biology [40,44]. To limit any confounding effects of ontogeny on comparative analyses in the present study, assessment focused on the morphologies and dietary habits of the largest size classes only (i.e., when intestinal length was most fully developed). Although the full range of items contributing to the diet of the examined terapontids have been quantified (22 different food classes [40]), in this study, gut contents were simply categorised as the percent contribution of animal material to species’ diet (i.e., the combined contribution of fish, insects and crustaceans). Arcsine transformations of dietary percentages were conducted prior to further analysis to improve normality [57].

### Body size-intestinal length correction

Appropriately correcting for body size effects and allometric scaling of morphological traits, while simultaneously taking phylogeny into account, poses an ongoing challenge for comparative studies [58]. To remove effects of body size and allometric scaling of intestinal length between terapontid species, the “phyl_resid” function outlined by Revell [58] was used to regress mean species’ intestinal lengths against mean standard lengths to produce phylogenetically size-corrected residuals in the R package “phytools” [59,60]. Hereafter, reference to intestinal length refers to the phylogenetically size-corrected estimate.

### Testing for phylogenetic signal

To test whether the traits considered in this study (intestinal length and volumetric plant-detrital proportions in diet) individually showed evidence of phylogenetic signal two metrics were utilised – the *K* statistic [61] and Pagel’s *λ*[62]. These statistics compare the observed fit of the data to the phylogeny with the analytical expectation based on the topology and branch lengths of the phylogeny, assuming a Brownian (random walk) model of character evolution. Blomberg’s *K* quantifies the amount of phylogenetic signal in the tip data relative to the expectation (*K* = 1) for a trait that evolved by Brownian motion along the specified topology and branch lengths [61]. Values of *K* close to 0 indicate random evolution of traits, values close to 1 correspond to a Brownian-motion-type evolution, and values < 1 indicate strong phylogenetic signal and trait conservatism. Following Blomberg [61], *K*'s significance was assessed using a data randomization test conducted by randomly permutating the tips of the phylogeny 1000 times. A significant phylogenetic signal was indicated if the observed *K* value was greater than across 95% of the randomizations.

Pagel’s *λ* provides the best fit of the Brownian motion model to the tip data by means of a maximum likelihood approach [63]. Thus, if *λ* = 1, the trait evolved according to the Brownian motion, and *λ* can take any value from 0 (i.e., a star phylogeny, where the trait shows no phylogenetic signal) to >1 (more phylogenetic signal than expected under the Brownian motion). The significance of *λ* can be assessed by a likelihood ratio comparison of nested models with particular values (i.e., 0 or 1).Tests for phylogenetic signal were implemented using the “phylosignal” and “Kcalc” functions in “phytools” [59]. Both statistics were calculated for traits based on the maximum clade credibility tree.

### Phylogenetic comparative analyses

Correlations between intestinal length and dietary composition were examined both with and without phylogenetic correction. To remove the possible correlation associated with phylogenetic relatedness, we calculated phylogenetically independent contrasts (PIC; [32]) of intestinal length and proportion of animal material in species’ diets. For PIC analysis, the molecular topology with branch lengths was imported into Mesquite 2.75 [51]. The PDAP (Phenotypic Diversity Analysis Package) module [64,65] implemented in Mesquite was used to calculate standardised independent contrasts for the correlation between size-corrected intestinal length and arcsine-transformed proportion of animal material in diet at 28 internal nodes on the terapontid phylogeny. The Pearson product-moment correlation coefficient *r* (computed through the origin) and its associated *P* value are reported. The relationship between the phylogenetically independent contrasts was then determined by using a reduced major axis regression (RMA) as there is considerable variation in calculation of both morphological and dietary variables.

Initial diagnostic plots of the absolute values of the standardized phylogenetically independent contrasts versus their standard deviations revealed that branch lengths of the phylogenetic tree adequately fitted the tip data, indicating that estimated branch lengths were adequate for the assumptions of independent contrasts [64]. While PIC is reasonably robust to violations of branch length assumptions [66], additional PICs were calculated using topologies with several arbitrary branch lengths as a sensitivity analysis for any potential uncertainty associated with branch lengths derived in the molecular phylogeny: branch lengths set to unity (1.0 – similar to a speciation model of character evolution), contemporaneous tips with internodes set to one [67], contemporaneous tips with internodes set to one less that the number of descendant tip species [68], and contemporaneous tips with internodes set to the log of number of descendant tip species [68]. All tree manipulation was done using Mesquite (version 2.75).

To assess the effects of failing to control for phylogenetic relatedness, a phylogenetically naive RMA regression (i.e., assuming a star phylogeny) was conducted to investigate the relationship between intestinal length residuals (calculated from an ordinary least squares regression of standard length versus intestinal length) versus arcsine-transformed proportion of animal material in diet.

## Results

### Phylogenetic analysis

**Figure 2:**
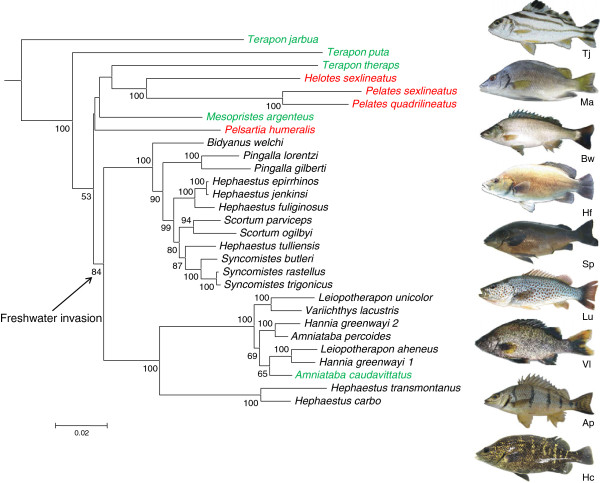
**Maximum likelihood phylogeny for 28 terapontid species based on analysis of combined nuclear and mitochondrial DNA.** All bootstrap values are based on 1000 pseudoreplicates. Outgroup species were pruned from the tree. Images are identified by initials of genus and species nearby in the tree. Taxon names are colour-coded according to macrohabitat associations identified in [44]: red = marine, green = euryhaline, and black = freshwater. The node signifying invasion of Australasian freshwater habitat is indicated.

Maximum likelihood recovered one tree with a likelihood score of -34413.698284 (Figure 2). Most nodes within the fresh water radiation in the tree were well resolved with strong support [69] evidenced by bootstrap values mostly >80. Marine-euryhaline species relationships mostly had no bootstrap support. A “highest clade credibility tree” generated from BEAST analyses also had a very similar structure to the ML approach, especially when considering well supported nodes (see Additional file 2: Figure S2), highlighting the general similarities in tree structure regardless of phylogenetic construction approach used.

### Dietary and morphological quantification

Data on diet, length and intestinal length of each species and trophic classifications from the literature are presented in Table 1. There is broad variability in terapontid diets, from those comprising only animal prey to those consuming almost no animal material. Presenting the often flexible dietary habits of fishes as averages can mask important spatio-temporal variability. Previous studies on the trophic ecology of terapontids (including specimens used in this study) identified low spatial and temporal dietary variability in many species, particularly herbivore-detritivores [70]. Dietary data derived in this study also agreed closely with often more seasonally and spatially comprehensive data from other sources and geographic areas [45,71,72]. This suggests available data should provide a robust approximation of typical diet for most species, particularly at the broad level of comparative amounts of animal prey in diet.

**Table 1:** Summary data on terapontid morphology and diet used in study

**Species**	***n***	**SL (mm)**	**IL (mm)**	**RIL (SL/IL) Range**	**% Animal prey**	**Trophic classification**
*Amniataba caudavittatus*	12 (14)	93.9 ± 27.8	85.4 ± 30.6	0.7–0.9	93	Invertivore
*Amniataba percoides*	28 (48)	110.5 ± 6.8	112.8 ± 15.0	0.8–1.2	44.2	Omnivore
*Bidyanus welchi*	9 (10)	204.1 ± 25.8	347.2 ± 36.8	1.6–2.2	70	Generalist carnivore
*Hannia greenwayi*	10 (19)	81.2 ± 25.7	74.0 ± 39.8	0.6–1.2	76.8	Invertivore
*Helotes sexlineatus*	36 (36)	123.4 ± 26.6	177.3 ± 23.2	1.3–1.7	22	Herbivore
*Hephaestus fuliginosus*	20 (42)	266.9 ± 23.1	556.3 ± 143.5	1.6–3.5	32.8	Omnivore
*Hephaestus carbo*	25 (27)	129.2 ± 13.6	126.7 ± 23.0	0.8–1.1	98.6	Invertivore
*Hephaestus epirrhinos*	3 (3)	223.7 ± 44.7	303.0 ± 93.3	1.2–1.5	80.4	Generalist carnivore
*Hephaestus jenkinsi*	22 (33)	195.9 ± 30.3	415.7 ± 83.2	1.5–2.8	45.1	Omnivore
*Hephaestus transmontanus*	20 (20)	76.8 ± 4.17	51.65 ± 5.18	0.6–0.8	99.6	Invertivore
*Hephaestus tulliensis*	14 (15)	171.5 ± 24.6	439.6 ± 90.5	1.7–3.0	23.3	Omnivore
*Leiopotherapon aheneus*	18 (25)	50.7 ± 9.7	96.3 ± 39.0	1.2–3.1	31.9	Herbivore
*Leiopotherapon unicolor*	30 (70)	136.8 ± 15.1	122.6 ± 20.1	0.8–1.2	91.1	Generalist carnivore
*Mesopristes argenteus*	13 (13)	156.7 ± 33.6	188.2 ± 63.8	0.9–1.4	96.2	Generalist carnivore
*Pelates quadrilineatus*	7 (7)	112.7 ± 17.0	106.6 ± 15.7	0.9–1.02	99.2	Generalist carnivore
*Pelates sexlineatus*	16 (16)	94.9 ± 15.2	85.1 ± 17.5	0.8–1.0	98.1	Generalist carnivore
*Pelsartia humeralis*	2 (2)	153.5 ± 7.78	142 ± 9.9	0.9–0.9	96	Generalist carnivore
*Pingalla gilberti*	29 (35)	67.5 ± 16.2	117.0 ± 30.2	1.2–2.3	17.4	Detritivore-algivore
*Pingalla lorentzi*	12 (12)	67.1 ± 18.7	122.5 ± 42.6	1.5–2.0	34	Detritivore-algivore
*Scortum ogilbyi*	17 (25)	275.0 ± 32.8	1297.6 ± 296.4	3.7–7.1	8	Herbivore
*Scortum parviceps*	28 (31)	264.0 ± 13.8	1427.8 ± 248.1	3.6–7.6	3	Herbivore
*Syncomistes butleri*	18 (21)	200.0 ± 18.9	786.5 ± 191.0	3.1–5.6	0.2	Detritivore-algivore
*Syncomistes rastellus*	12 (13)	108.4 ± 38.6	415.4 ± 294.1	3.0–6.4	7.7	Detritivore-algivore
*Syncomistes trigonicus*	23 (26)	71.9 ± 16.3	232.2 ± 123.9	3.0–5.3	2.7	Detritivore-algivore
*Terapon jarbua*	26 (20)	106.0 ± 29.4	111.4 ± 22.7	0.9–1.2	99	Generalist carnivore
*Terapon puta*	6 (6)	131.2 ± 35.5	125.7 ± 37.6	0.9–1.0	96.4	Generalist carnivore
*Terapon theraps*	8 (8)	148.9 ± 14.1	144.8 ± 20.2	0.9–1.1	99.9	Generalist carnivore
*Variichthys lacustris*	11 (11)	150.4 ± 34.8	141.1 ± 82.0	0.7–1.1	47	Omnivore

Study species, specimen numbers (*n*), mean values (±S.D.) for each species’ morphological measurements, relative intestinal length (RIL) range, percentage animal material in diet, and trophic classification. *n* signifies the number of intestinal length measurements per species, with the sample numbers used to derive dietary data in parentheses. Trophic classifications sourced from [44].

Relative intestinal length (IL/SL) is the most commonly used descriptor in diet-morphology assessments [17], so RIL ranges are provided for comparison with published data (Table 1). Reduced major axis regressions of log_10_–transformed standard length versus log_10_–transformed intestinal length for each species over the available studied size are also outlined in supporting information (see Additional file 3: Table S1).

### Ontogenetic development of intestinal morphology

**Figure 3:**
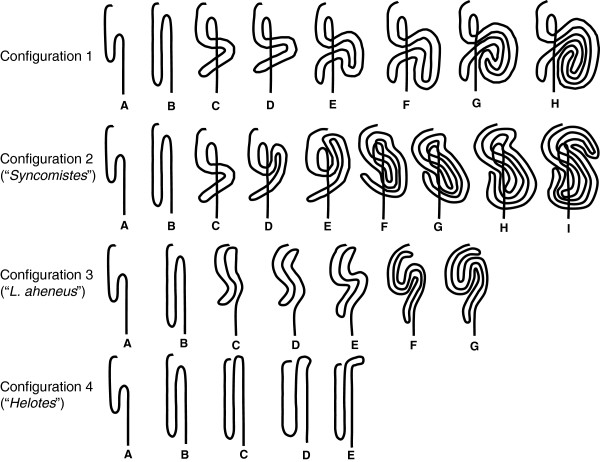
**Patterns of ontogenetic development of intestinal layout in terapontids.** Intestinal tracts are viewed ventrally, the anterior-most portion of the intestine (outlet of the pylorus) is always located to the top of each figure.

Fish digestive tracts were examined for elaborations such as hindgut chambers, caecal pouches and valves. The only external differences in intestinal structure between terapontid species appear to be length and coiling patterns. The simplest intestinal layout consisted of two loops and was evident immediately after post-larval metamorphosis in all species examined (Figure 3, configuration 1A-1B). The first loop occurred posterior of the pylorus near the rear of the body cavity, after a slight dextral curve immediately posterior to the pylorus. The intestine continued anteriorly until a second loop occurred ventral to the stomach, after which the intestine continued posteriorly to the anus, producing an “s-shaped or two-loop” layout. This simple configuration was evident throughout the life history of *Leiopotherapon unicolor*, *Amniataba percoides*, *A. caudavittatus*, *Hannia greenwayi*, *Hephaestus carbo*, *Hep. epirrhinos*, *Hep. transmontanus*, *Pelates sexlineatus*, *P. quadrilineatus*, *Terapon theraps*, *T. puta*, *T. jarbua* and *Varichthys lacustris* (see Additional file 2: Figure S3); however, significant allometric increases in intestinal length were achieved in several species by increasing the length of each loop in both anterior and posterior directions (Figure 3, configuration 1B). RILs of <1.2 typify terapontid species that retained the two-loop intestinal configuration throughout their life history (Table 1). The “two-loop” intestinal configuration was the juvenile morphology of all remaining species.

In all other species except *L. aheneus* and *Hel. sexlineatus*, a transverse folding and elongation of the middle portion of the two-loop intestinal pattern occurred, directing the elongated section to the left of the body cavity (Figure 3, configuration 1C and 1D) and ventrally beneath the posteriorly directed section of the two-loop pattern. This produced the “six-loop” configuration described by Vari [42], which was the layout throughout the remaining life history of *Bidyanus welchi*, *Hep. fuliginosus*, *Hep. jenkinsi* and *Hep. tulliensis* (see Additional file 2: Figure S4). Adult RILs of ~2 to 2.5 characterized these species (Table 1). In *Pingalla* and *Scortum* species, the loops on the right-hand side of the body cavity continued dorso-anteriorly before turning to lengthen in a posterior direction (Figure 3, configuration 1E-1F). In *Pingalla* species this remained the intestinal layout of adults. A further increase in intestinal complexity occurred in *Scortum* species and was characterised by additional convolution in a spiral configuration (Figure 3, configuration 1G-1H). In all of these species the majority of convolution occurred on the left-hand side of the body cavity. The RILs of *Scortum* species averaged ~4.5, and reached over 7 in some specimens (Table 1; Additional file 2: Figure S7).

A different development of intestinal configuration was evident in *Syncomistes* species. Like *Hephaestus*, *Pingalla* and *Scortum* species, *Syncomistes* species developed the “six-loop” pattern, but the subsequent looping in *Syncomistes* proceeded anteriorly before folding and lengthening to the right-hand side of the body cavity. At the same time, posterior looping from the “six-loop” configuration proceeded to the left-hand side of the body cavity behind the stomach (Figure 3, configuration 2C-2E). This was followed by a reversal of looping directions in both the anterior and posterior sections, looping back to the left- and right-hand side of the body cavity respectively (Figure 3, configuration 2F- 2I). This complex intestinal configuration resulted in RILs of *Syncomistes* reaching over 6 in some specimens (Table 1; Additional file 2: Figure S10).

Another distinct pattern of ontogenetic intestinal looping was evident in *Leiopotherapon aheneus*. From the initial two-loop pattern the anterior loop lengthened anteriorly along the ventral surface of the stomach close to the pyloric outlet (Figure 3, configuration 3A-3B). This was followed by a folding in the middle section of the intestine (Figure 3, configuration 3C-3E). This folding initially proceeded anteriorly along the dorso-ventral plane of the body before turning to the right-hand side of the body cavity (Figure 3, configuration 3F-3G). The majority of folding in this pattern occurred on the right-hand side of the body. Intestinal lengths of *L. aheneus* typically reached between 2-3 times standard length in larger specimens (Table 1; Additional file 2: Figure S8).

A final distinct pattern of ontogenetic intestinal looping was evident in *Hel. sexlineatus.* From the initial two-loop pattern, the posterior and anterior loops extended in both directions during ontogeny. The anterior loop then extended past the pyloric outlet, before looping around the anterior aspect of the stomach, crossing the dorso-ventral plane to lengthen into the anterior, right-hand side of the body cavity (Figure 3, configuration 4D-4E). While only a comparatively minor increase in complexity, this configuration produced higher RILs compared to the standard “two-loop” intestinal layout (Table 1; Additional file 2: Figure S11).

### Character optimisations and reconstruction of ancestral character states

**Figure 4:**
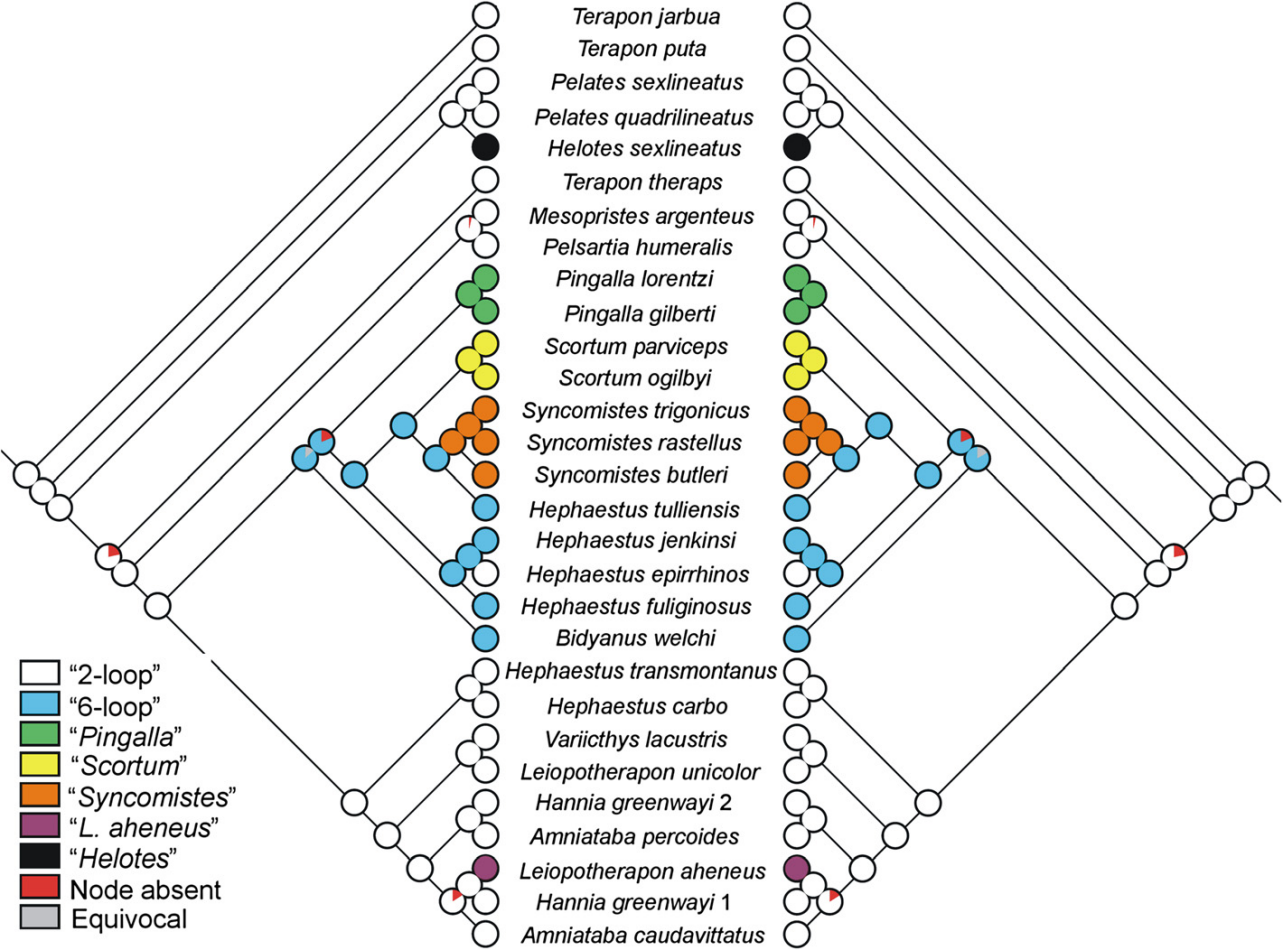
**Ancestral character state reconstruction of terapontid intestinal morphology.** Summary of maximum likelihood (left graph) and maximum parsimony (right) ancestral character reconstruction of adult intestinal configuration for 450 terapontid trees displayed on the maximum clade credibility tree. Circles at terminal nodes represent the observed character state for extant species. Pie charts for ancestral nodes show estimated proportions for reconstructed character states at that internal node.

Optimising adult intestinal configuration patterns onto the maximum clade credibility phylogeny indicated that the ontogenetic development of increased intestinal complexity has evolved independently on three occasions in terapontid fishes. While a range of patterns of ontogenetic increase in intestinal complexity have evolved in the clade containing *Hephaestus*, *Scortum*, *Bidyanus*, *Syncomistes* and *Pingalla* species, ontogenetic increases in intestinal convolution were limited to just a single species (*L. aheneus*) in the other major freshwater clade, as well as on a single occasion in the euryhaline/marine clade (*Hel. sexlineatus*). An examination of ancestral state reconstructions across the 450 trees from the BEAST analysis yielded very similar predictions between parsimony and likelihood analyses (Figure 4) and the inferred ancestral states for terapontid intestinal length configuration were not substantially affected by uncertainty in tree topology, branch lengths, or character state reconstruction methods. Both MP and ML analysis indicated that the “two-loop” pattern is unequivocally plesiomorphic within Terapontidae, and that the “two-loop” intestinal pattern was exhibited by the most recent common ancestor of all freshwater species (i.e., at the time of fresh water invasion). Both reconstruction approaches also indicated that the evolution of adult intestinal complexity followed a complex pattern of multiple independent gains and one loss within both major freshwater clades. Both approaches indicated that the “six-loop” intestinal configuration was a precursor to the range of more complex intestinal patterns evident in *Pingalla*, *Scortum* and *Syncomistes* species. Character state reconstruction also suggested that the two similar patterns of increase evident in *Pingalla* and *Scortum* species evolved independently. An apparent reversion to the plesiomorphic state of an adult “two-loop” intestinal pattern was also evident in *Hep. epirrhinos*, the only species within this clade to retain this intestinal configuration as an adult.

### Phylogenetic signal

Blomberg’s *K* and Pagel’s *λ* for proportion of animal prey in diet and intestinal length both demonstrated significant levels of phylogenetic signal, indicating that neither variable was independent and, therefore, phylogenetic comparative methods were justified in further analyses. While the estimates of phylogenetic signal for the two variables were both significant, the patterns of phylogenetic signal were not convergent. Phylogeny was a significant predictor of variation in animal material in terapontid diet (*K* = 0.73, observed PIC variance = 1.01, *P* < 0.001, Pagel’s *λ* = 0.88, *P* < 0.001). However, both *K* and *λ* were estimated to be considerably less than 1, suggesting a phylogenetic signal lower than the one expected under Brownian motion and, accordingly, substantial evolutionary lability in terapontid diet, even between closely related species. Phylogeny accounted for a larger component of variability in intestinal length in the terapontids (*K* = 1.05, observed PIC variance = 0.294, *P* < 0.001, Pagel’s *λ* = 0.94, *P* < 0.001), suggesting a phylogenetic signal close to what would be expected under Brownian motion in both statistics.

### Comparative analyses

**Figure 5:**
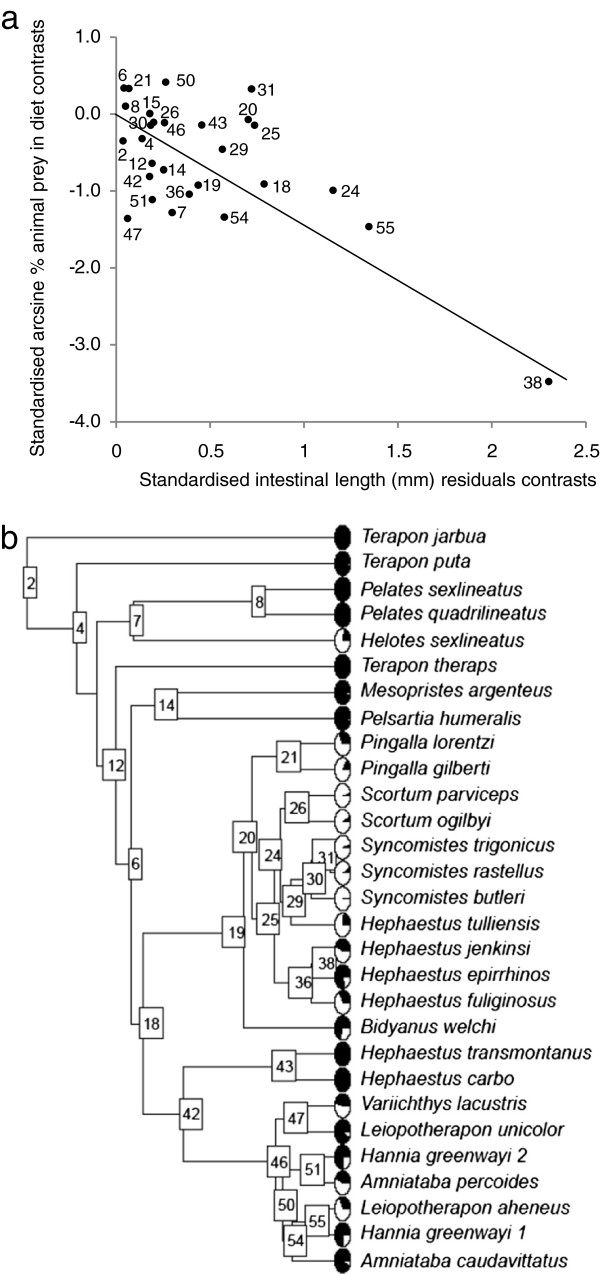
**Phylogenetic independent contrasts of terapontid diet versus intestinal length.** (**a**) Relationship between phylogenetically independent contrasts of intestinal length residuals and contrasts of arcsine transformed proportion of animal material in diet. Numbers represent the nodes (contrasts) indicated in the phylogeny in (**b**).

After correcting for phylogenetic proximity, the independent contrasts of intestinal length versus diet were significantly, and negatively, correlated with the percentage of animal material in terapontid diet, explaining 65% of variation in diet composition (*r*^*2*^= 0.65, RMA slope = -1.48, *P* < 0.001). Twenty-two of the 28 independent contrasts were negative, and occurred across both deep and shallow nodes of the phylogeny (Figure 5). Several of the most negative contrasts occurred at nodes within the phylogeny (nodes 38, 55, 24, 29, 7, 18 and 19) that were precursors to gains/losses in intestinal complexity identified in the character mapping and ancestral character reconstruction (Figure 4). This highlights the importance of gains in intestinal complexity in facilitating dietary radiation. PICs with branch lengths set to unity (*r*^*2*^ = 0.46, RMA slope = -1.94, *P* < 0.001), Nee in Purvis [68] branch lengths (*r*^*2*^ = 0.52, RMA slope = -1.80, *P* < 0.001), Grafen [73] branch lengths (*r*^*2*^ = 0.54, RMA slope = -1.71, *P* < 0.001) and Pagel’s [67] branch lengths (*r*^*2*^ = 0.49, RMA slope = -1.82, *P* < 0.001) all produced similar results to the molecular phylogeny. A phylogenetically naive RMA regression also identified a significant negative relationship between intestinal length residuals and arcsine-transformed proportion of animal material in diet, and, furthermore, this analysis explained a greater proportion of data variation than any of the phylogenetic comparative analyses (*r*^*2*^ = 0.72, RMA slope = -1.50, *P* < 0.001).

## Discussion

### Evolution of intestinal length and dietary radiation in terapontids

Several patterns of ontogenetic development of increased intestinal length were evident in the terapontid species examined. Like previous studies [41-43], results highlighted an underlying phylogenetic component to these developmental patterns. The interspecific differences in intestinal length resulting from these ontogenetic developmental mechanisms explained a significant amount of the variability in the volume of animal prey in terapontid diets. Results indicate that the widely held ecomorphological maxim of longer digestive tracts equating with increasing consumption of non-animal prey, holds true for terapontids, even when accounting for phylogenetic relationships between species. Study outcomes align with a growing number of studies, where if phylogeny is taken into account, carnivores have shorter intestines than related species consuming larger amounts on non-animal prey [25,26].

This study produced a number of commonalities as well as contrasts to the previous work on the family outlined in Vari [42]. Both studies identified the “two-loop” intestinal configuration as being the plesiomorphic adult pattern within Terapontidae. This study suggested a number of different contrasts to the patterns of intestinal development across the family, at both species and family levels. The secondary loss of the “six-loop” intestinal layout Vari [42] proposed in “*Hephaestus* genus b” instead appears due to the polyphyly of *Hephaestus* and phylogenetic location of this “*Hephaestus* genus b” in a separate clade of species with a “two-loop” intestinal layout. Vari [42] suggested that *Scortum* species shared the same adult “six-loop” intestinal pattern as *Bidyanus* and *Hephaestus* species (Figure 1). The current studyinstead highlighted *Scortum* and *Syncomistes* species as developing the most complex intestinal patterns of any terapontid species. This study also identified previously undescribed pattern of ontogenetic intestinal length increase in *L. aheneus* and *Hel. octolineatus.* The different topology emerging from molecular relationships compared to Vari’s [42] phylogeny also suggested a different sequence of intestinal length complexity across the family. Rather than the progressive increase in complexity as genera become more derived, proposed by Vari [42] (Figure 1), a more complex historical process of development was predicted from molecular relationships. Character-state reconstruction inferred that the relatively complex intestinal configurations of adult *Pingalla*, *Scortum* and *Syncomistes* species all evolved from the six-loop pattern on three separate occasions. The novel ontogenetic development documented in both *L. aheneus* and *Hel. octolineatus* also demonstrated that the capacity for significantly increasing intestinal length during ontogeny has evolved independently in both major clades of freshwater terapontids as well as euryhaline-marine species. These multiple independent origins of increased intestinal complexity across several clades suggests convergent evolution toward increased intestinal length in terapontids having diets with lower proportions of animal prey.

Although the role of ontogenetic phenomena in phyletic evolution remains strongly debated [74-76], modification of ontogenetic development is proposed as one of the most common mechanisms through which morphological change and novelties originate during phyletic evolution [74,75]. The development of intestinal complexity in terapontids exhibits several elements of heterochronic processes [74,75], where ontogeny is modified to produce morphological novelty. Several possible peramorphic (recapitulatory) processes, for example, could explain the apparent repetition of adult intestinal layouts (two-loop and six-loop patterns) of ancestral forms during the ontogeny of many descendent terapontid taxa, before additional intestinal complexity is added to ancestral configurations. A range of associated heterochronic processes (acceleration, hypermorphosis and pre-displacement) can all produce descendent phenotypes that transcend the ancestral form [74,75]. Similarly, paedomorphic phenomena, where adults retain the juvenile morphology of putative ancestral taxa, could explain the apparent retention of two-loop intestinal layout throughout the life history of *Hep. epirrhinos*, within a clade of closely related *Hephaestus* species that have a six-loop configuration (Figure 4). Without a range of additional size/age and possibly shape-based data on terapontid ontogenetic trajectories [71,77], the exact role of heterochronic processes can only be speculated upon. However, recapitulation does appear to be a recurrent theme in the development of intestinal length complexity in a number of fish lineages [43]. With additional genetic and morphological data, terapontids may provide a valuable model lineage for elucidating the role of modification of ontogeny as a driver of evolutionary diversification.

### The utility of intestinal length as a predictor of diet

While standard regression and PIC approaches both highlighted significant relationships between intestinal length and animal material in the diet, the amount of variability explained was lower in the PIC analysis. This difference underlines the importance of comparative methods in not overstating the strength of the association between morphology and ecology [27]. Although intestinal length emerged from the phylogenetically informed analysis as a useful predictor of diet, a substantial amount of unexplained variability was also evident in the relationship. Behavioural, ecological, physiological and historical factors can interact to influence the strength of the congruence between morphological and ecological characters [78]. Issues associated with age, phenotypic plasticity, antecedent food availability (i.e., periods of starvation) as well as the relative levels of different dietary substrates have emerged from both field and controlled laboratory studies as possibly inducing changes in intestinal length [17,33,79,80]. While intestinal length is clearly a somewhat plastic character, ontogenetic and phylogenetic factors appear more influential than diet on gut dimensions in some fish clades [33], suggesting a precedence of genetic adaptation over phenotypic plasticity as the major force acting on the digestive system. Intestinal looping patterns identified in this study were largely consistent with previous research (see Vari; [42]), and seemed species/genus-specific, but strength of any underlying genetic component to their expression needs to be tested with controlled feeding experiments e.g., [33,79]. The capacity for at least some phenotypic plasticity in intestinal length in response to different trophic opportunities could promote initial divergence in dietary habits, and potentially provide scope for natural selection to extend and consolidate the phenotypic response.

While intestinal length may be a useful predictor of broad dietary habits, it may have a variable capacity to predict finer scale dietary divisions among omnivores [28]. Many of the terapontids examined here consume varying proportions of both animal and plant or detrital material, and would require more robust dietary and morphological data to adequately test ecomorphological relationships at a finer scale. Omnivory has been interpreted as a compromise strategy in which protein from scarce animal prey is complemented by energy from abundant primary foods [81]. Omnivory and generalist diets are also regarded as an adaptive response to seasonal variations in water level and trophic resources that characterise hydrologically variable tropical river systems [82]. With the wet-dry tropical catchments that harbour the majority of terapontid diversity ranking among some of the most hydrologically variable globally, versatility in feeding habits is, not unexpectedly, a common feature of many terapontid diets [44,45].

We also used intestinal length as a dietary predictor in relation to stomach content data. Classifying diets on the basis of stomach content analysis can be problematic for fishes, particularly nominal herbivores and detritivores, dietary habits expressed by several species in this study (at least on the basis of stomach content data). Conventional macroscopic dietary quantification can be prone to inadequately identifying the actual nutritional targets of ingestion, and often require integration with microscopic, histological or stable isotopic approaches to accurately define dietary ecology. Many marine ‘herbivores’ once commonly perceived to be algivores have been revealed by detailed dietary analyses to be highly dependent on amorphic detritus scraped from epilithic algal complexes [16,83,84]. Similarly, recent studies have indicated that freshwater ‘detritivorous’ fishes assimilate carbon from biofilm and seston, and nitrogen from intermediate microbial decomposers in the environment, and are not capable of direct assimilation of vascular plant carbon [16]. In contrast to the abundant research on terrestrial vertebrate ‘nutritional ecology’, the nutritional targets, food composition and associated digestive functioning of herbivorous-detritivorous fish are poorly defined [10,18,84]. While these gaps are being addressed in the marine environment (incrementally in some areas; [84]), they are equally, if not more pronounced in freshwater species, and pose a considerable impediment to understanding the trophic ecology and food web function of herbivorous-detritivorous freshwater fishes [16,85,86].

Alimentary anatomy is frequently an unreliable indicator of functional capacity of herbivorous fishes, particularly if the digestive tract is considered in isolation [84]. Morphological and functional changes to the biomechanics and musculoskeletal functional morphology related to food procurement and handling are considered critical components in the impressive evolutionary diversification and ecological success of teleosts, including many herbivorous and detritivorous fishes [87,88]. There are marked changes in oral anatomy (flattened, depressible dentition, dentary rotation etc.) across several of the freshwater genera within the Terapontidae, such as *Scortum*, *Pingalla* and *Syncomistes* species, that have adopted diets volumetrically dominated by plant and/or detrital material Figure 1; [42]. Interestingly, the marine herbivore *Hel. sexlineatus*, recently separated from the *Pelates* genus [89], also appears to have evolved flattened, tricuspidite dentition similar to that of freshwater herbivores [90]. Assessment of these changes to oral anatomy and feeding kinematics in relation to terapontid trophic diversification would be a valuable complement to the role of intestinal modification documented in this study.

Intestinal length considered in isolation is also in many ways a simplistic indicator of the functional morphology of fish intestinal tracts. Other aspects of digestive morphology and physiology such as intestinal diameter, digesta passage rates, ultra-structural surface area and digestive enzyme profiles can also have significant associations with diet [24,25,91-94]. Beyond the significant correlation between increasing intestinal length and decreasing animal prey in diet, interpretation of the evolution of specific dietary habits in the Terapontidae must be treated with caution as we currently have limited insights into the physiological processes associated with extracting and utilizing nutrients from consumed foods. A nutritional ecological approach, however, incorporating knowledge of diet, functional morphology, intake, digestive physiology and dietary assimilation [sensu 18,86] would provide a more robust foundation with which to resolve the trophic habits of terapontids. Regardless of the underlying nutritional targets and associated digestive mechanisms, however, the pronounced shifts toward non-animal prey evident in many terapontids are clearly associated with significant modification of intestinal length.

### Terapontids as a model system for studying dietary diversification

The capacity to increase intestinal length, and associated shifts away from carnivory, have evolved independently across multiple marine-euryhaline and freshwater genera within Terapontidae, but are especially pronounced in freshwater species. Shifts away from carnivory and evolution of herbivory and plant-detrital diets are prominent in many of the more speciose and ecologically diverse marine and freshwater fish lineages, often marking a profound shift in the phylogenetic trajectories, species diversity and ecological impact of certain clades [87,95]. The significant diet-intestinal length relationship evident in approximately 55% of extant terapontid species suggests that the capacity to develop long intestines during ontogeny has facilitated the widespread shifts away from carnivorous diets across the family.

Studies of trophically diverse lineages using cladistics and assessment of digestive tract characters could be useful in elucidating the process of evolution of herbivorous-detritivorous trophic habits [17]. Terapontids could provide such a model to demonstrate the process of evolution of non-animal prey-based diets from an ancestrally carnivorous lineage. With carnivory the likely ancestral habit of the euryhaline-marine ancestors of Australia’s freshwater terapontids, the invasion of fresh waters saw adoption of a variety of omnivorous, herbivorous and detritivorous dietary habits during the terapontid fresh water radiation [45]. Due to its biogeographic isolation the Australian freshwater fish fauna is particularly unusual for its prevalence of acanthopterygian percomorph fishes (which typically dominate marine habitats), and for an almost complete lack of ostariophysan fishes which dominate fresh water environments on other continents. The timing of Australia’s break-up from Gondwana precluded the presence of cichlids, characiforms, cypriniformes and most siluriformes, which represent the dominant proportion of herbivores and detritivores in other continents’ freshwater fish faunas [95,96]. The majority of Australia’s freshwater fishes are ‘secondary’ freshwater teleostean species (i.e., freshwater species derived from marine ancestors), often with strong affinities to tropical Indo-Pacific marine taxa [97,98].

Fossil evidence suggests that the Terapontidae has had a long evolutionary history (≥ 40-45 Ma) in Australian fresh waters [99]. Paleoecological conditions that may have facilitated the dietary diversification of early fresh water-invading terapontids, particularly shifts away from carnivory, probably include a range of vacant niches due to a lack of an incumbent herbivorous-detritivorous fish fauna [45]. Similar processes and timescales relating to ecological opportunity and release from competitive constraints have been proposed to explain the significant morphological disparification and lineage diversification evident in Australasian ariid catfishes following a similar fresh water invasion [100]. Following invasion of a new habitat, species may show a rapid burst of cladogenesis and associated ecomorphological (often diet-related) diversification [3,101-103]. The majority of morphological divergence in characters like intestinal convolution and dentition appear to have occurred independently on several occasions in freshwater terapontids [43]; this study. The significant relationship between intestinal length and shifts away from animal prey in the diet of terapontids suggests that the evolution of longer intestines, in particular, facilitated much of the dietary diversification evident in Australian fresh water environments.

## Conclusions

Intestinal length is a significant correlate to interspecific dietary variation in terapontids. The ontogenetic development of intestinal complexity appears to represent an important functional innovation driving much of the ecological (trophic) radiation evident within Terapontidae. The significant negative correlation between trophic morphology (intestinal length) and proportion of animal material in terapontid diet suggests resource-based divergent selection as an important diversifying force in the adaptive radiation of Australia’s freshwater terapontids, particularly the pronounced shifts away from ancestral carnivorous dietary habits evident across the family. Much previous research has suggested that modifications of oral anatomy and functional associations with initial food procurement are one of the primary drivers of fish lineage diversification [36,37,104,105]. The capacity to modify intestinal morphology-physiology in light of new digestive challenges may also be an important facilitator of trophic diversification during phyletic radiations see also [8,26,106]. Moreover, the ontogenetic development of a range of intestinal convolutions being limited to freshwater terapontids is suggestive of ecomorphological character release within the family following invasion of fresh waters by ancestral euryhaline-marine species. Assessment of the relative patterns of lineage diversification between freshwater and euryhaline-marine terapontids in other aspects of trophic morphology sensu [100] and ecology would be fruitful avenues for research on the phylogenetic effects of adaptive zone shifts.

## Competing interests

The authors declare that they have no competing interests.

## Authors’ contributions

AMD conceptualized the study and conducted field and laboratory work and carried out the phylogenetic analyses. PJU developed the molecular phylogenetic trees. BJP and DLM conducted field work and specimen collection. AMD, PJU, BJP, RGP and DLM wrote the paper. All authors read and approved the final manuscript.

## Supplementary Material

Additional file 1: Figure S1 Image of maximum likelihood tree for Terapontidae species derived in Davis et al. [45]. The maximum likelihood tree (-ln = -36324.681391) for Terapontidae species derived in Davis et al. (2012b), based on a combined analysis of cytochrome b and the recominbination activation 1 and 2 gene sequences (5952 bp). Species highlighted in bold indicate those utilised in the current comparative study. Bootstrap values are presented as ML/MP, with an # representing nodes with support from both methods > 99. Click here for file

Additional file 2: Figure S2 Bayesian *BEAST species tree for Terapontidae based on analysis of the mitochondrial cytochrome b gene and the combined nuclear recombination activation genes 1 and 2. The analysis was based on 50 million generations, with parameters logged every 5000 generations with a burn-in of 10%. The posterior probability is shown to the right of each node. **Figure S3-S12**. Images of terapontid intestinal morphology development. Images of terapontid intestinal morphology. Click here for file

Additional file 3: Table S1 Terapontid intestinal length scaling analyses. Results for scaling analyses of reduced major axis regressions of Log_10_ –transformed standard length versus Log_10_ – transformed intestinal length for 27 terapontid species. Statistically significant allometric scaling relationship (i.e., where the 95% confidence interval for slope does not overlap with an isometric slope of 1.0) are highlighted in bold. n signifies the number of intestinal length measurements per species. Click here for file

## References

[B1] Grant PR Ecology and Evolution of Darwin’s Finches 1986 Princeton: Princeton University Press

[B2] Albertson RC Markert JA Danley PD Kocher TD Phylogeny of a rapidly evolving clade: the cichlid fishes of Lake Malawi, East Africa Proc Natl Acad Sci USA 1999 96 5107 5110 10.1073/pnas.96.9.5107 10220426 PMC21824

[B3] Streelman JT Danley PD The stages of vertebrate evolutionary radiation TREE 2003 18 126 131

[B4] Vitt LJ Pianka ER Cooper WE Schwenk K History and global ecology of squamate reptiles Am Nat 2003 162 44 60 10.1086/375172 12856236

[B5] Clements KD Raubenheimer D Evans DH, Claiborne JB Feeding and nutrition The Physiology of Fishes 2006 3 Gainesville: CRC Press 47 82

[B6] Espinoza RE Wiens JJ Tracy CR Recurrent evolution of herbivory in small, cold climate lizards: breaking the ecophysiological rules of reptilian herbivory Proc Natl Acad Sci USA 2004 101 16819 16824 10.1073/pnas.0401226101 15550549 PMC534712

[B7] Burbrink FT Pyron RA How does ecological opportunity influence rates of speciation, extinction and morphological diversification in New World ratsnakes (Tribe Lampropeltini)? Evolution 2010 64 934 943 19895554 10.1111/j.1558-5646.2009.00888.x

[B8] Herrel A Huyghe K Vanhooydonck B Backeljau T Breugelmans K Grbac I Van Damme R Irschick DJ Rapid large‐scale evolutionary divergence in morphology and performance associated with exploitation of a different dietary resource Proc Natl Acad Sci USA 2008 105 4792 4795 10.1073/pnas.0711998105 18344323 PMC2290806

[B9] Price SA Hopkins SSB Smith KK Roth VL Tempo of trophic evolution and its impact on mammalian diversification Proc Natl Acad Sci USA 2012 109 7008 7012 10.1073/pnas.1117133109 22509033 PMC3345017

[B10] Choat JH Clements KD Vertebrate herbivores in marine and terrestrial environments: a nutritional ecology perspective Ann Rev Ecol Syst 1998 29 375 403 10.1146/annurev.ecolsys.29.1.375

[B11] Knoppel HA Food of central Amazonian fishes Amazonia 1970 2 257 352

[B12] Lowe-McConnell RH Fish communities in tropical freshwaters 1975 London: Longmans

[B13] Horn MH Evans DH Feeding and digestion The Physiology of Fishes 1998 2 Boca Raton: CRC Press 43 63

[B14] Horn MH Ojeda FP Horn MH, Martin KLM Herbivory Intertidal Fishes: Life in Two Worlds 1999 San Diego: Academic 197 222

[B15] Nelson JS Fishes of the World 2006 Hoboken: John Wiley and Sons

[B16] Lujan NK German DP Winemiller KO Do wood grazing fishes partition their niche? Morphological and isotopic evidence for trophic segregation in neotropical Loricariidae Func Ecol 2011 25 1327 1338 10.1111/j.1365-2435.2011.01883.x

[B17] Horn MH Biology of marine herbivorous fishes Ocean Mar Biol Ann Rev 1989 27 167 272

[B18] Karasov WH, Martínez del Rio C Physiological Ecology: How Animals Process Energy, Nutrients, and Toxins 2007 Princeton: Princeton University Press

[B19] German DP Farrell AP, Cech JJ, Richards JG, Stevens ED Digestive efficiency Encyclopedia of Fish Physiology, From Genome to Environment 2011 San Diego: Elsevier 1596 1607

[B20] Karasov WH Martínez del Rio C Caviedes-Vidal E Ecological physiology of diet and digestive systems Ann Rev Phys 2011 73 69 93 10.1146/annurev-physiol-012110-142152 21314432

[B21] Sibly RM Townsend CR, Calow P Strategies of digestion and defecation Physiological Ecology: an Evolutionary approach to Resource use 1981 Oxford: Blackwell Scientific Publications 109 139

[B22] Stevens CE Hume ID Comparative Physiology of the Vertebrate Digestive System 2nd Edition 1995 Melbourne: Press Syndicate of the University of Cambridge

[B23] Ricklefs RE Morphometry of the digestive tracts of some Passerine birds The Condor 1996 98 279 292 10.2307/1369146

[B24] Elliott JP Bellwood DR Alimentary tract morphology and diet in three coral reef fish families J Fish Biol 2003 63 1598 1609 10.1111/j.1095-8649.2003.00272.x

[B25] German DP Nagle BC Villeda JM Ruiz AM Thompson AW Bald SC Evans DH Evolution of herbivory in a carnivorous clade of minnows (Teleostei: Cyprinidae): effects on gut size and digestive physiology Physiol Biochem Zool 2010 83 1 18 10.1086/648510 19929637

[B26] Wagner CE McIntyre PB Buels KS Gilbert DM Michael E Diets predict intestine length in Lake Tanganyika’s cichlid fishes Func Ecol 2009 23 1122 1131 10.1111/j.1365-2435.2009.01589.x

[B27] Berumen ML Pratchett MS Goodman BA Relative gut lengths of coral reef butterflyfishes (Pisces: Chaetodontidae) Coral Reefs 2011 30 1005 1010 10.1007/s00338-011-0791-x

[B28] Kramer DL Bryant MJ Intestine length in fishes of a tropical stream: 2. Relationships to diet – the long and short of a convoluted issue Env Biol Fishes 1995 45 129 141

[B29] Horn MH Gawlicka A German DP Logothetis EA Cavanagh JW Boyle KS Structure and function of the stomachless digestive system in three related species of New World silverside fishes (Atherinopsidae) representing herbivory, omnivory, and carnivory Mar Biol 2006 149 1237 1245 10.1007/s00227-006-0281-9

[B30] Choat JH Robbins WD Clements KD The trophic status of herbivorous fishes on coral reefs – II: Food processing modes and trophodynamics Mar Biol 2004 145 445 454

[B31] Day RD German DP Tibbetts IR Why can’t young fish eat plants? Neither digestive enzymes nor gut development preclude herbivory in the young of a stomachless marine herbivorous fish Comp Biochem Physiol B 2010 158 23 29 20884371 10.1016/j.cbpb.2010.09.010

[B32] Felsenstein J Phylogenies and the comparative method Am Nat 1985 125 1 15 10.1086/284325

[B33] German DP Horn MH Gut length and mass in herbivorous and carnivorous prickleback fishes (Teleostei: Stichaeidae): ontogenetic, dietary, and phylogenetic effects Mar Biol 2006 148 1123 1134 10.1007/s00227-005-0149-4 15547797

[B34] Herrel A Meyers JJ Nishikawa KC De Vree F The evolution of feeding motor patterns in lizards: modulatory complexity and possible constraints Am Zool 2001 41 1311 1320 10.1668/0003-1569(2001)041[1311:TEOFMP]2.0.CO;2

[B35] Herrel A Podos J Vanhooydonck B Hendry AP Force-velocity trade-off in Darwin’s finch jaw function: a biomechanical basis for ecological speciation? Func Ecol 2009 23 119 125 10.1111/j.1365-2435.2008.01494.x

[B36] Westneat MW Evolution of levers and linkages in the feeding mechanisms of fishes Integr Comp Biol 2004 44 378 389 10.1093/icb/44.5.378 21676723

[B37] Higham TE Hulsey CD Rican O Carroll AM Feeding with speed: prey capture evolution in cichlids J Evol Biol 2007 20 70 78 10.1111/j.1420-9101.2006.01227.x 17210001

[B38] Pfennig DW Wund MA Snell-Rood EC Cruickshank T Schlichting CD Moczek AP Phenotypic plasticity’s impacts on diversification and speciation TREE 2010 25 459 467 20557976 10.1016/j.tree.2010.05.006

[B39] Kramer DL Bryant MJ Intestine length in the fishes of a tropical stream: 1. Ontogenetic allometry Environ Biol Fishes 1995 42 115 127 10.1007/BF00001990

[B40] Davis AM Pusey BJ Pearson RG Trophic ecology of terapontid grunters: the role of morphology and ontogeny Mar Freshw Res 2011 63 128 141

[B41] Zihler F Gross morphology and configuration of digestive tracts of cichlidae (Teleostei, Perciformes): phylogenetic and functional significance Netherlands J Zool 1982 32 544 571

[B42] Vari RP The terapon perches (Percoidei: Teraponidae): a cladistic analysis and taxonomic revision Bull Am Mus Nat Hist 1978 159 175 340

[B43] Yamaoka K Intestinal coiling pattern in epilithic algal-feeding cichlids (Pisces, Teleostei) of Lake Tanganyika, and its phylogenetic significance Zool J Linn Soc 1985 84 235 261 10.1111/j.1096-3642.1985.tb01800.x

[B44] Davis AM Pearson RG Pusey BJ Perna C Morgan DL Burrows D Trophic ecology of northern Australia’s terapontids: ontogenetic dietary shifts and feeding classification J Fish Biol 2011 78 265 286 10.1111/j.1095-8649.2010.02862.x 21235560

[B45] Davis AM Unmack PJ Pusey BJ Pearson RG Marine-freshwater transitions are associated with the evolution of dietary diversification in terapontid grunters (Teleostei: Terapontidae) J Evol Biol 2012 25 1163 1179 10.1111/j.1420-9101.2012.02504.x 22519660

[B46] Katoh K Toh H Parallelization of the MAFFT multiple sequence alignment program Bioinformatics 2010 26 1899 1900 10.1093/bioinformatics/btq224 20427515 PMC2905546

[B47] Zwickl DJ Genetic algorithm approaches for the phylogenetic analysis of large biological sequence datasets under the maximum likelihood criterion. PhD Thesis 2006 Austin: University of Texas

[B48] Posada D Crandall KA Modeltest: testing the model of DNA substitution Bioinformatics 1998 14 817 818 10.1093/bioinformatics/14.9.817 9918953

[B49] Swofford DL PAUP*. Phylogenetic Analysis Using Parsimony (* and other methods), Version 4.0b10 2003 Sunderland: Sinauer

[B50] Yagishita N Miya M Yamanoue Y Shirai SM Nakayama K Suzuki N Mitogenomic evaluation of the unique facial nerve pattern as a phylogenetic marker within the perciform fishes (Teleostei: Percomorpha) Mol Phyl Evol 2009 53 258 266 10.1016/j.ympev.2009.06.009 19540351

[B51] Maddison WP Maddison DR Mesquite: A Modular System for Evolutionary Analysis, ver. 2:75 2011 http://mesquiteproject.org

[B52] Drummond AJ Rambaut A BEAST: Bayesian evolutionary analysis by sampling trees BMC Evol Biol 2007 7 214 10.1186/1471-2148-7-214 17996036 PMC2247476

[B53] Schluter D Price T Mooers AØ Ludwig D Likelihood of ancestor states in adaptive radiation Evolution 1997 51 1699 1711 10.2307/2410994 28565128

[B54] Pagel M The maximum likelihood approach to reconstructing ancestral character states of discrete characters on phylogenies Syst Biol 1999 48 612 622 10.1080/106351599260184

[B55] Lewis PO A likelihood approach to estimating phylogeny from discrete morphological character data Syst Biol 2001 50 913 925 10.1080/106351501753462876 12116640

[B56] Mooers AØ Schluter D Reconstructing ancestral states with maximum likelihood: support for one- and two-rate models Syst Biol 1999 48 623 633 10.1080/106351599260193

[B57] Sokal RR Rohlf FJ Biometry: The Principles and Practice of Statistics in Biological Research 1995 3 New York: W. H. Freeman and Co.

[B58] Revell LJ Size-correction and principal components for interspecific comparative studies Evolution 2009 63 3258 3268 10.1111/j.1558-5646.2009.00804.x 19663993

[B59] Revell LJ phytools: an R package for phylogenetic comparative biology (and other things) Methods Ecol Evol 2013 3 217 223 10.1002/ece3.448

[B60] R Development Core Team R: a language and environment for statistical computing 2011 Vienna, Austria: R Foundation for Statistical Computing http://www.R-project.org

[B61] Blomberg SP Garland T Jr Ives AR Testing for phylogenetic signal in comparative data: behavioral traits are more labile Evolution 2003 57 717 745 12778543 10.1111/j.0014-3820.2003.tb00285.x

[B62] Pagel M Inferring the historical patterns of biological evolution Nature 1999 401 877 884 10.1038/44766 10553904

[B63] Freckleton RP Harvey PH Pagel M Phylogenetic analysis and comparative data: a test and review of evidence Am Nat 2002 160 712 726 10.1086/343873 18707460

[B64] Garland T Jr Harvey PH Ives AR Procedures for the analysis of comparative data using phylogenetically independent contrasts Syst Biol 1992 41 18 32

[B65] Midford PE Garland T Jr Maddison WP PDAP Package, version 1.15 2010 http://mesquiteproject.org/pdap_mesquite/

[B66] Garland T Midford PE Ives AR An introduction to phylogenetically based statistical methods, with a new method for confidence intervals on ancestral values Am Zool 1999 39 374 388

[B67] Pagel MD A method for the analysis of comparative data J Theor Biol 1992 156 431 442 10.1016/S0022-5193(05)80637-X

[B68] Purvis A A composite estimate of primate phylogeny Philos Trans R Soc Lond B 1995 348 405 421 10.1098/rstb.1995.0078 7480112

[B69] Hillis DM Bull JJ An empirical test of bootstrapping as a method for assessing confidence in phylogenetic analysis Syst Biol 1993 42 182 192

[B70] Davis AM Pearson RG Pusey BJ Contrasting intraspecific dietary shifts in two terapontid assemblages from Australia’s wet-dry tropics Ecol Freshw Fish 2011 21 42 56

[B71] Bishop KA Allen SA Pollard DA Cook MG Ecological Studies on the Fishes of the Alligator Rivers Region, Northern Territory: Autecology Office of the Supervising Scientist Report 145 2001 Canberaa Australia: Supervising Scientist

[B72] Storey AW A review of dietary data from fish of the Fly River System: A precursor to constructing a food web. A report prepared for Ok Tedi Mining Ltd 1998 Perth: Wetland Research and Management, Western Australia

[B73] Grafen A The phylogenetic regression Philos Trans R Soc Lond B 1989 326 119 157 10.1098/rstb.1989.0106 2575770

[B74] Gould SJ Ontogeny and phylogeny 1977 Cambridge: Harvard University Press

[B75] Alberch P Gould SJ Oster GF Wake DB Size and shape in ontogeny and phylogeny Paleobiology 1979 5 296 317

[B76] Webster M Zelditch ML Evolutionary modifications of ontogeny: heterochrony and beyond Paleobiology 2005 31 354 372 10.1666/0094-8373(2005)031[0354:EMOOHA]2.0.CO;2

[B77] Sues HD Evolution of herbivory in terrestrial vertebrates: perspectives from the fossil record 2000 Cambridge: Cambridge University Press

[B78] Motta PJ Norton SF Luczkovich JL Perspectives on the ecomorphology of bony fishes Environ Biol Fishes 1995 44 11 20 10.1007/BF00005904

[B79] Olsson J Quevedo M Colson C Svanback R Gut length plasticity in perch: into the bowels of resource polymorphisms Biol J Linn Soc 2007 90 517 523 10.1111/j.1095-8312.2007.00742.x

[B80] Davis AM Pusey BJ Trophic polymorphism and water clarity in northern Australian Scortum (Pisces: Terapontidae) Ecol Freshw Fish 2010 19 638 643 10.1111/j.1600-0633.2010.00448.x

[B81] Bowen SH Lutz EV Ahlgren MO Dietary protein and energy as determinants of food quality: trophic strategies compared Ecology 1995 76 899 907 10.2307/1939355

[B82] Lowe-McConnell RH Fish Communities in Tropical Freshwaters 1975 London: Longman Press

[B83] Crossman DJ Choat JH Clements KD Nutritional ecology of nominally herbivorous fishes on coral reefs Mar Ecol Prog Ser 2005 296 129 142

[B84] Clements KD Raubenheimer D Choat JH Nutritional ecology of marine herbivorous fishes: ten years on Func Ecol 2009 29 79 92

[B85] Mill AC Pinnegar JK Polunin NVC Explaining isotope trophic-step fractionation: why herbivorous fish are different Func Ecol 2007 21 1137 1145 10.1111/j.1365-2435.2007.01330.x

[B86] Davis AM Blanchette ML Pusey BJ Pearson RG Jardine TD Gut-content and stable-isotope analyses provide complementary understanding of ontogenetic dietary shifts and trophic relationships among fishes in a tropical river Freshwater Biol 2012 57 2156 2172 10.1111/j.1365-2427.2012.02858.x

[B87] Bellwood DR Origins and escalation of herbivory in fishes: a functional perspective Paleobiology 2003 29 71 83 10.1666/0094-8373(2003)029<0071:OAEOHI>2.0.CO;2

[B88] Cooper WJ Parsons K McIntyre A Kern B McGee-Moore A Bentho-pelagic divergence of cichlid feeding architecture was prodigious and consistent during multiple adaptive radiations within African rift-lakes PLoS One 2010 5 e9551 10.1371/journal.pone.0009551 20221400 PMC2833203

[B89] Mees GF Kailola PJ The freshwater Therapontidae of New Guinea Zool Verhand 1977 153 3 88

[B90] Sun BL A new species of Teraponidae from China Acta Zool Sin 1991 37 254 257

[B91] Al-Hussaini AH On the functional morphology of the alimentary tract of some fish in relation to differences in their feeding habits: anatomy and histology Quart J Microscop Sci 1949 90 109 139 18132292

[B92] Hofer R Morphological adaptations of the digestive tract of tropical cyprinids and cichlids to diet J Fish Biol 1988 33 399 408 10.1111/j.1095-8649.1988.tb05481.x

[B93] Frierson EW Foltz JW Comparison and estimation of Absorptive Intestinal Surface areas in two species of Cichlid Fish Trans Am Fish Soc 1992 121 517 523 10.1577/1548-8659(1992)121<0517:CAEOAI>2.3.CO;2

[B94] Tibbetts IR The distribution and function of mucous cells and their secretions in the alimentary tract of Arrhamphus sclerolepis krefftii J Fish Biol 1997 50 809 820

[B95] Barlow GW The cichlid fishes 2000 Cambridge: Perseus Publishing

[B96] Schaefer SA Lauder GV Historical transformation of functional design: evolutionary morphology of feeding mechanisms in Loricarioid catfishes Syst Zool 1986 35 498 508

[B97] Lundberg JG Kottelat M Smith GR Stiassny MLJ Gill AC So many fishes, so little time: an overview of recent ichthyological discovery in continental waters Ann Missour Botan Gard 2000 87 26 62 10.2307/2666207

[B98] Allen GR Midgley SH Allen M 2002: Field Guide to the Freshwater Fishes of Australia 2002 Perth: Western Australian Museum, Western Australia

[B99] Turner S A catalogue of fossil fish in Queensland Mem Queensl Mus 1982 20 599 611

[B100] Betancur-R R Ortí G Stein AM Marceniuk AP Pyron RA Apparent signal of competition limiting diversification after ecological transitions from marine to freshwater habitats Ecol Lett 2012 15 822 830 10.1111/j.1461-0248.2012.01802.x 22672567

[B101] Schluter D The ecology and origin of species TREE 2001 16 372 380 11403870 10.1016/s0169-5347(01)02198-x

[B102] Blondel J Evolution and ecology of birds on islands: trends and prospects Vie et Milieu 2000 50 205 220

[B103] Schluter D The Ecology of Adaptive Radiation 2000 Oxford: Oxford University Press

[B104] Schaefer B Rosen DE Major adaptive levels in the Actinopterygian feeding mechanism Am Zool 1961 1 187 204

[B105] Lauder GV Patterns of evolution in the feeding mechanism of actinopterygian fishes Am Zool 1982 20 275 285

[B106] Konow N Bellwood DR Functional disparity and ecological diversification in Marine Angelfishes, f. Pomacanthidae PLoS One 2011 6 e24113 10.1371/journal.pone.0024113 21909414 PMC3164712

