# Supplementary material for: Ontogenetic development of intestinal length and relationships to diet in an Australasian fish family (Terapontidae)
Source: BMC Evol Biol. 2013 Feb 25;13:53. doi: 10.1186/1471-2148-13-53 (PMC3598832; doi:10.1186/1471-2148-13-53)
Supplement: Additional file 1: Figure S1 — Image of maximum likelihood tree for Terapontidae species derived in Davis et al. [45]. The maximum likelihood tree (-ln = -36324.681391) for Terapontidae species derived in Davis et al. (2012b), based on a combined analysis of cytochrome b and the recominbination activation 1 and 2 gene sequences (5952 bp). Species highlighted in bold indicate those utilised in the current comparative study. Bootstrap values are presented as ML/MP, with an # representing nodes with support from both methods > 99. [file 1471-2148-13-53-S1.pptx]

## Slide 1
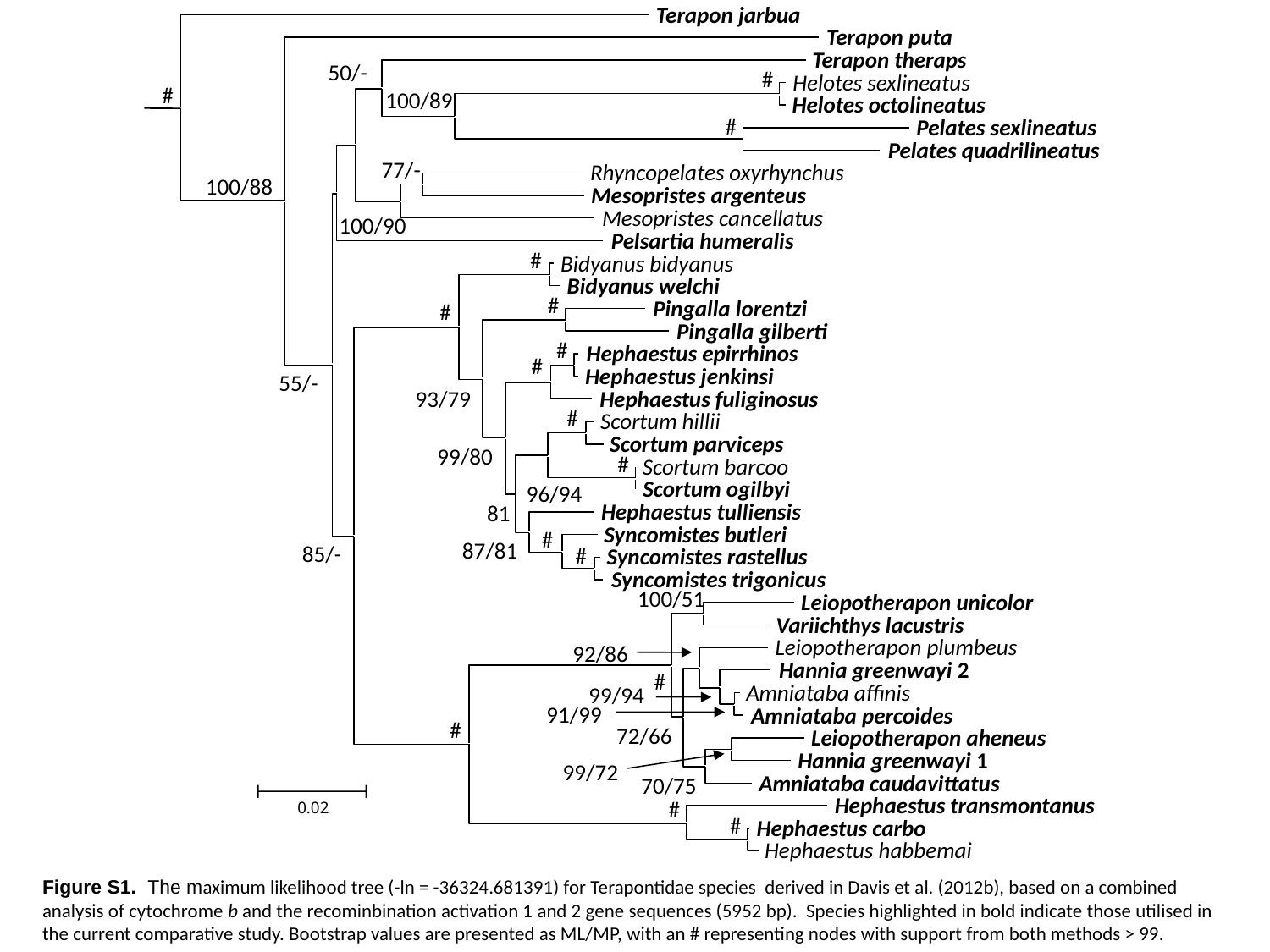

Terapon jarbua
 Terapon puta
 Terapon theraps
50/-
#
 Helotes sexlineatus
#
100/89
 Helotes octolineatus
#
 Pelates sexlineatus
 Pelates quadrilineatus
77/-
 Rhyncopelates oxyrhynchus
100/88
 Mesopristes argenteus
 Mesopristes cancellatus
100/90
 Pelsartia humeralis
#
 Bidyanus bidyanus
 Bidyanus welchi
#
 Pingalla lorentzi
#
 Pingalla gilberti
#
 Hephaestus epirrhinos
#
 Hephaestus jenkinsi
55/-
93/79
 Hephaestus fuliginosus
#
 Scortum hillii
 Scortum parviceps
99/80
#
 Scortum barcoo
 Scortum ogilbyi
96/94
 Hephaestus tulliensis
81
 Syncomistes butleri
#
87/81
85/-
#
 Syncomistes rastellus
 Syncomistes trigonicus
100/51
 Leiopotherapon unicolor
 Variichthys lacustris
 Leiopotherapon plumbeus
92/86
 Hannia greenwayi 2
#
 Amniataba affinis
99/94
91/99
 Amniataba percoides
#
72/66
 Leiopotherapon aheneus
 Hannia greenwayi 1
99/72
 Amniataba caudavittatus
70/75
0.02
 Hephaestus transmontanus
#
#
 Hephaestus carbo
 Hephaestus habbemai
Figure S1. The maximum likelihood tree (-ln = -36324.681391) for Terapontidae species derived in Davis et al. (2012b), based on a combined analysis of cytochrome b and the recominbination activation 1 and 2 gene sequences (5952 bp). Species highlighted in bold indicate those utilised in the current comparative study. Bootstrap values are presented as ML/MP, with an # representing nodes with support from both methods > 99.
