# Supplementary material for: Ontogenetic development of intestinal length and relationships to diet in an Australasian fish family (Terapontidae)
Source: BMC Evol Biol. 2013 Feb 25;13:53. doi: 10.1186/1471-2148-13-53 (PMC3598832; doi:10.1186/1471-2148-13-53)
Supplement: Additional file 2: Figure S2 — Bayesian *BEAST species tree for Terapontidae based on analysis of the mitochondrial cytochrome b gene and the combined nuclear recombination activation genes 1 and 2. The analysis was based on 50 million generations, with parameters logged every 5000 generations with a burn-in of 10%. The posterior probability is shown to the right of each node. Figure S3-S12. Images of terapontid intestinal morphology development. Images of terapontid intestinal morphology. [file 1471-2148-13-53-S2.pdf]

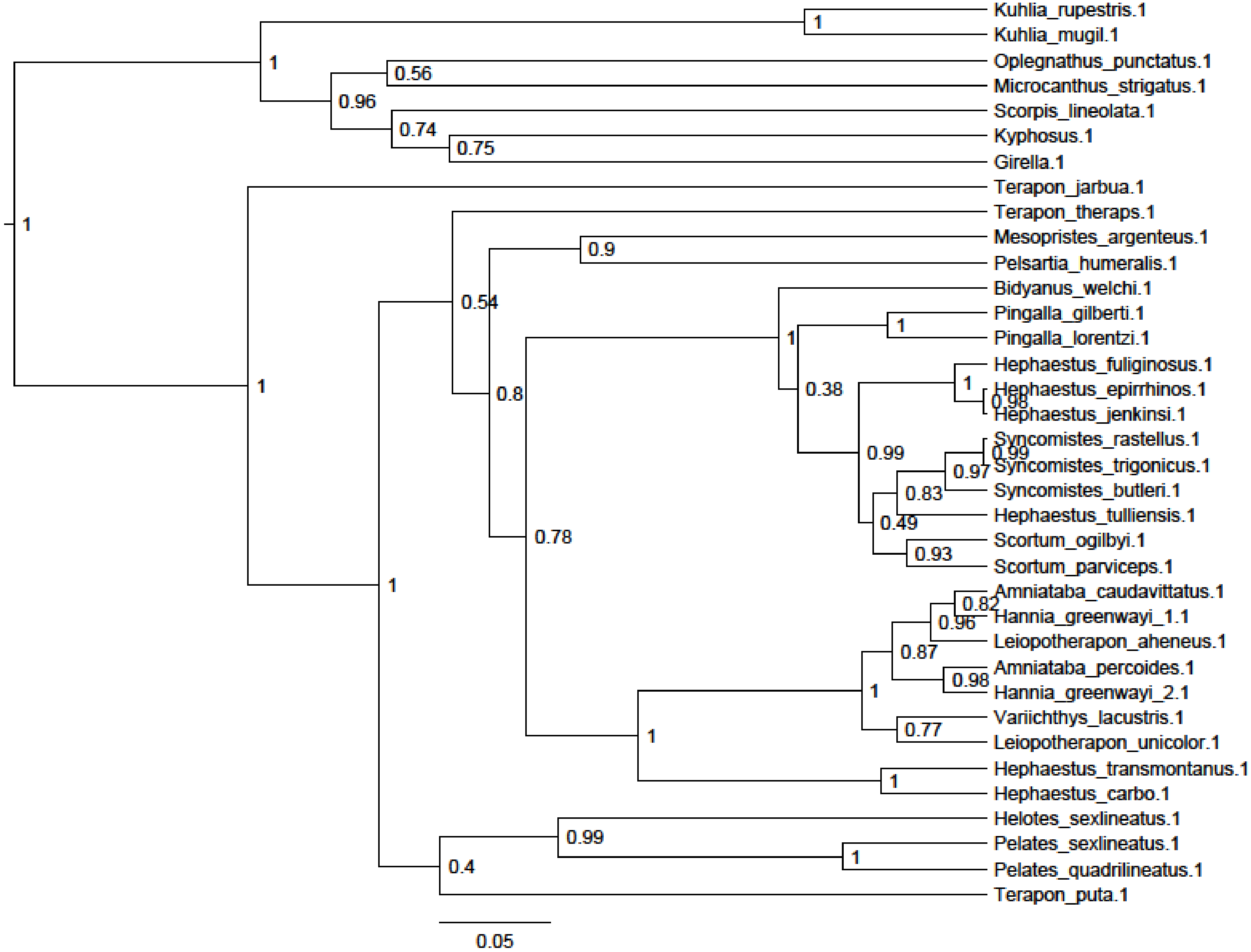

Figure S2. Bayesian *BEAST species tree for Terapontidae based on analysis of the mitochondrial cytochrome b gene and the combined nuclear recombination activation genes 1 and 2. The analysis was based on 50 million generations, with parameters logged every 5000 generations with a burn-in of 10%. The posterior probability is shown to the right of each node.

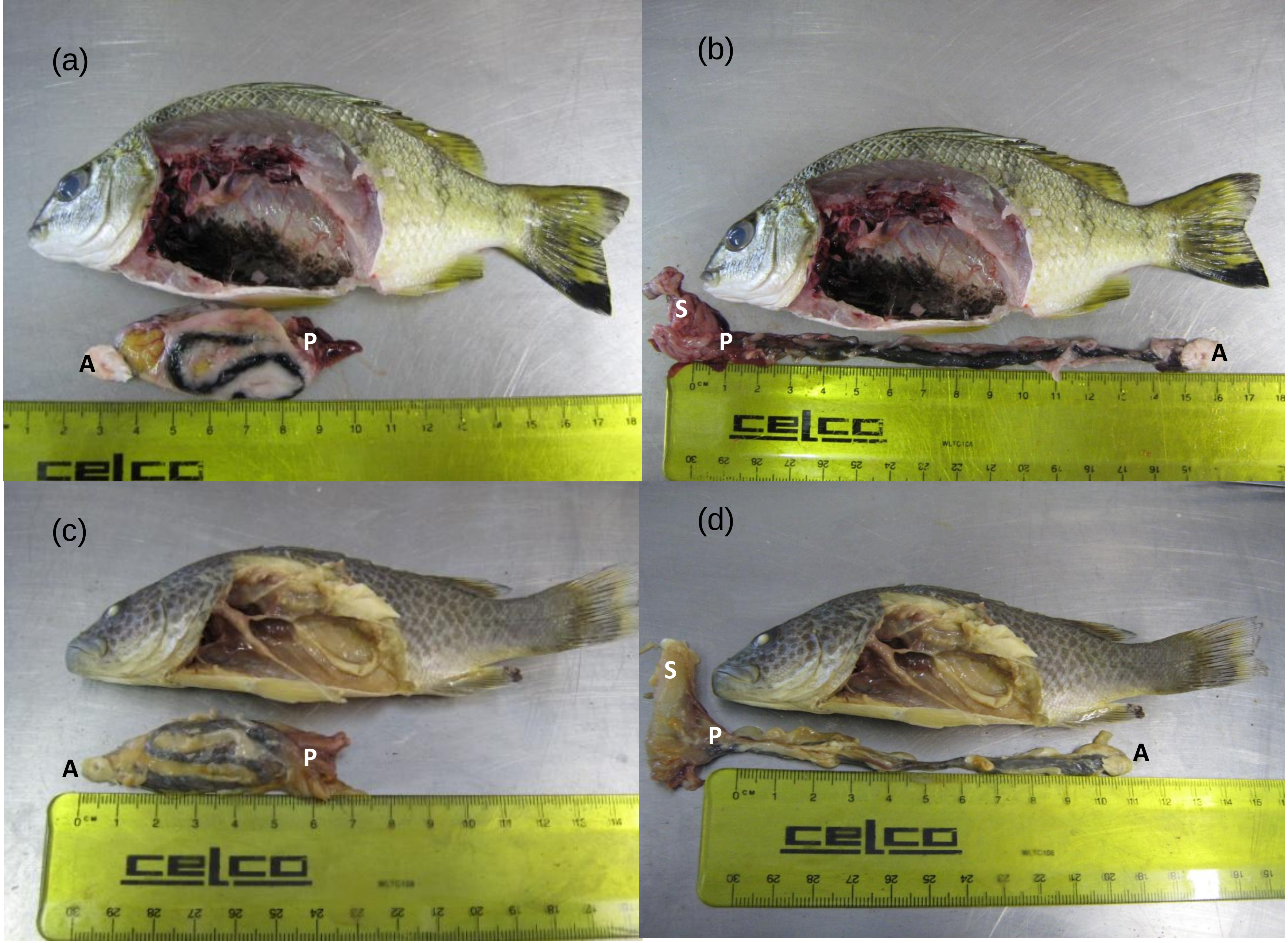

Figure S3. Terapontid intestinal morphology. (a) *Amniataba percoides* adult, “two-loop” intestine, excised and rotated; (b) *Amniataba percoides* intestine fully extended. (c) *Leiopotherapon unicolor* adult, “two-loop” intestine, excised and rotated; (d) *Leiopotherapon unicolor* intestine fully extended. S, stomach; P, pylorus; A, anus.

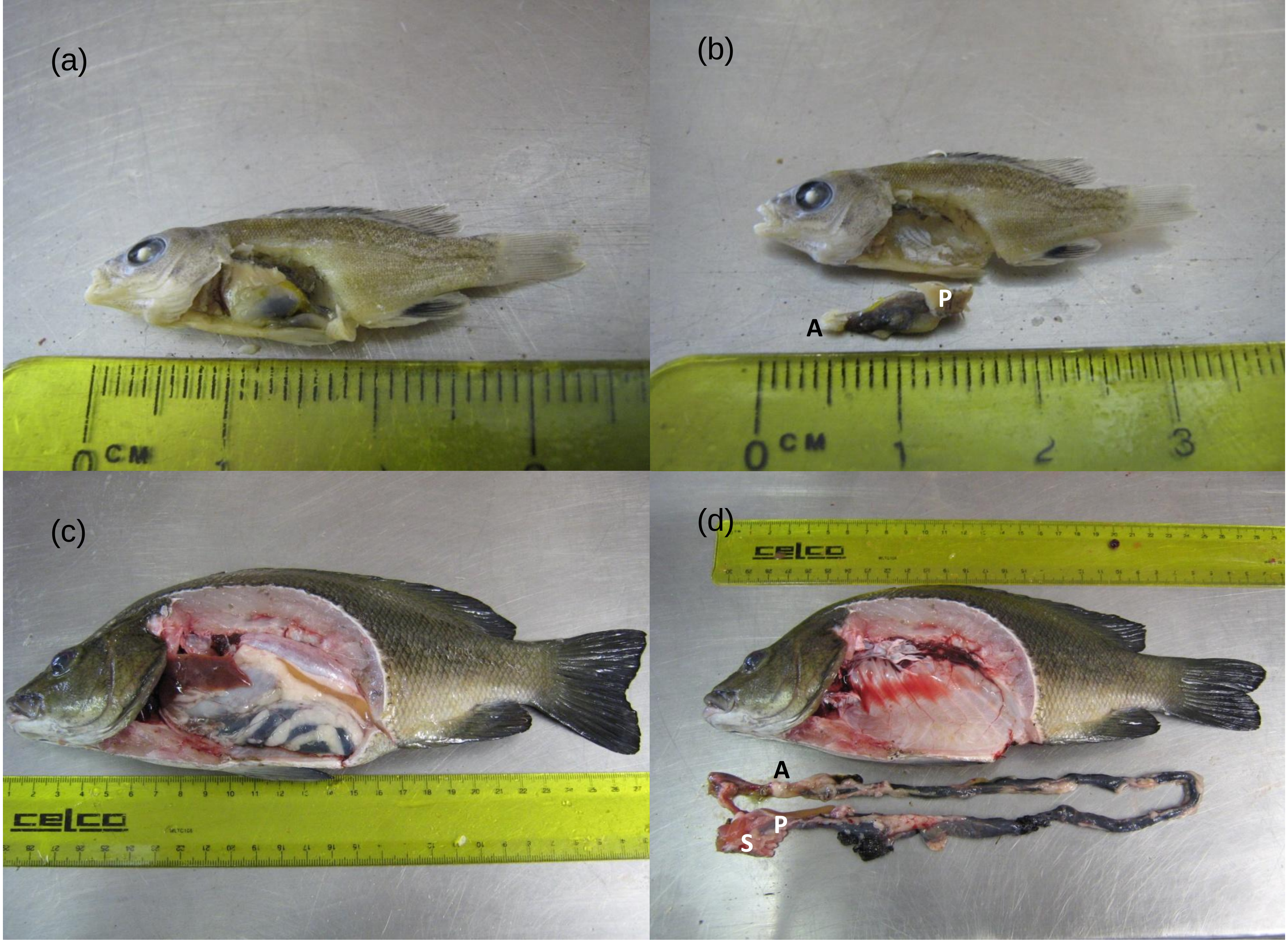

Figure S4. Terapontid intestinal morphology. (a) *Hephaestus fuliginosus* juvenile, “two-loop” intestine in situ; (b) *Hephaestus fuliginosus* juvenile intestine, excised and rotated; (c) *Hephaestus fuliginosus* adult, “six-loop” intestine in situ; (d) *Hephaestus fuliginosus* intestine extended. S, stomach; P, pylorus; A, anus.

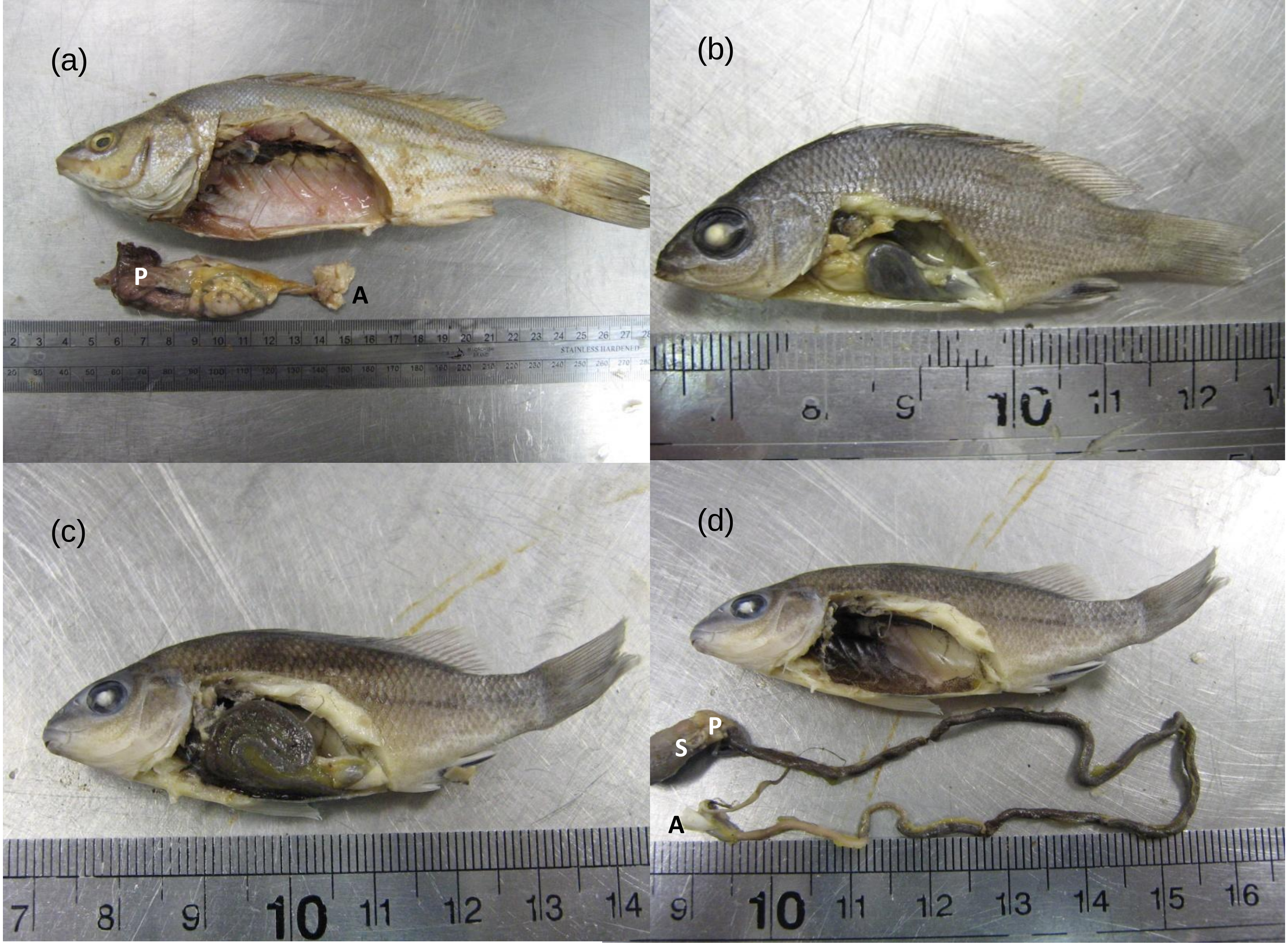

Figure S5. Terapontid intestinal morphology. (a) *Bidyanus welchi* adult, “six-loop” intestine, excised; (b) *Pingalla gilberti* sub-adult “six-loop” intestine in situ; (c) *Pingalla gilberti* adult, “*Pingalla*” intestine in situ; (d) *Pingalla gilberti* adult intestine extended. S, stomach; P, pylorus; A, anus.

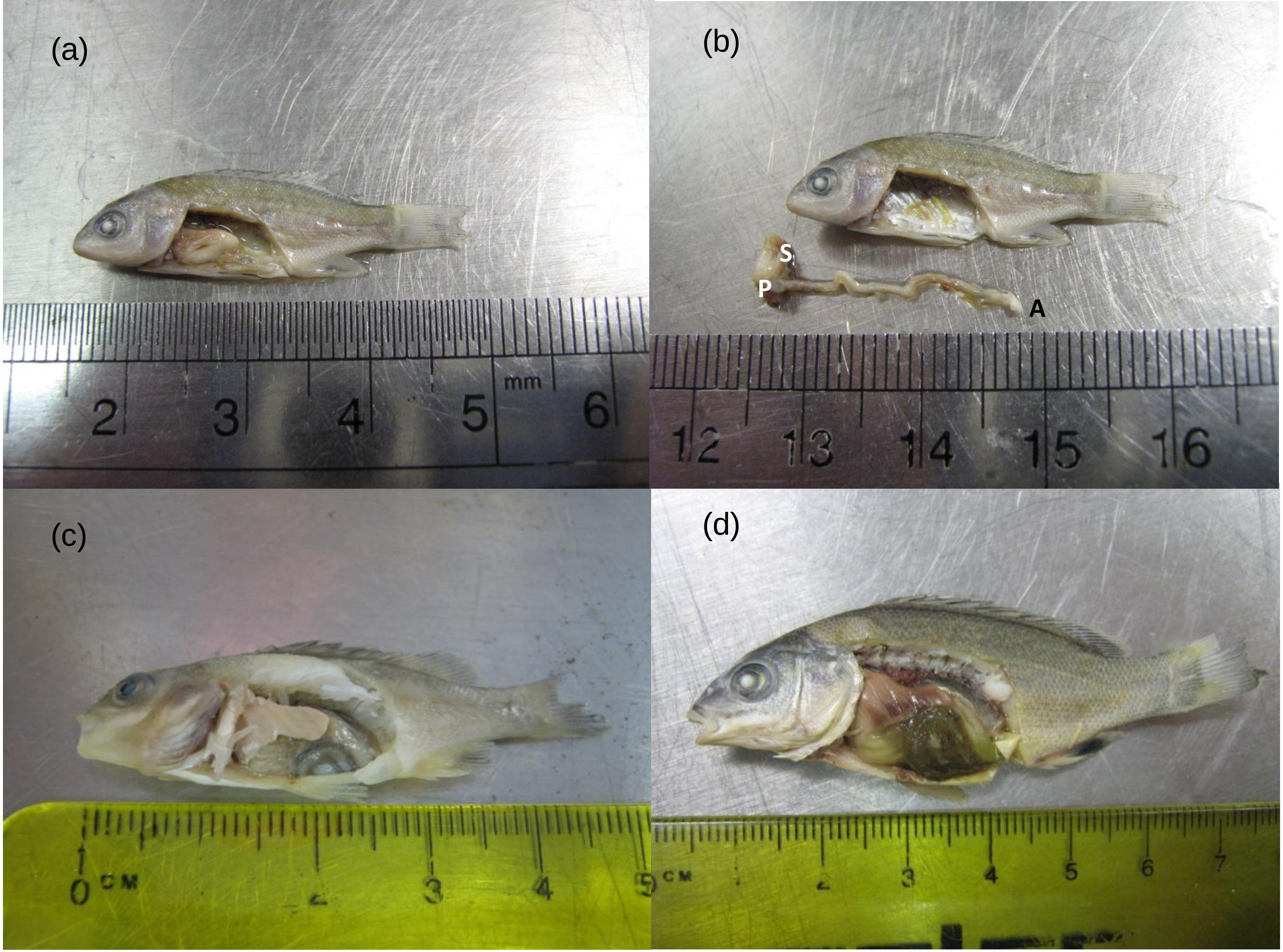

Figure S6. Terapontid intestinal morphology. (a) *Scortum parviceps* juvenile, “two-loop” intestine in situ; (b) *Scortum parviceps* juvenile, “six-loop” intestine fully extended (c) *Scortum parviceps* juvenile, early “six-loop” intestine in situ; (d) *Scortum parviceps* sub-adult, late “six-loop” intestine in situ . S, stomach; P, pylorus; A, anus.

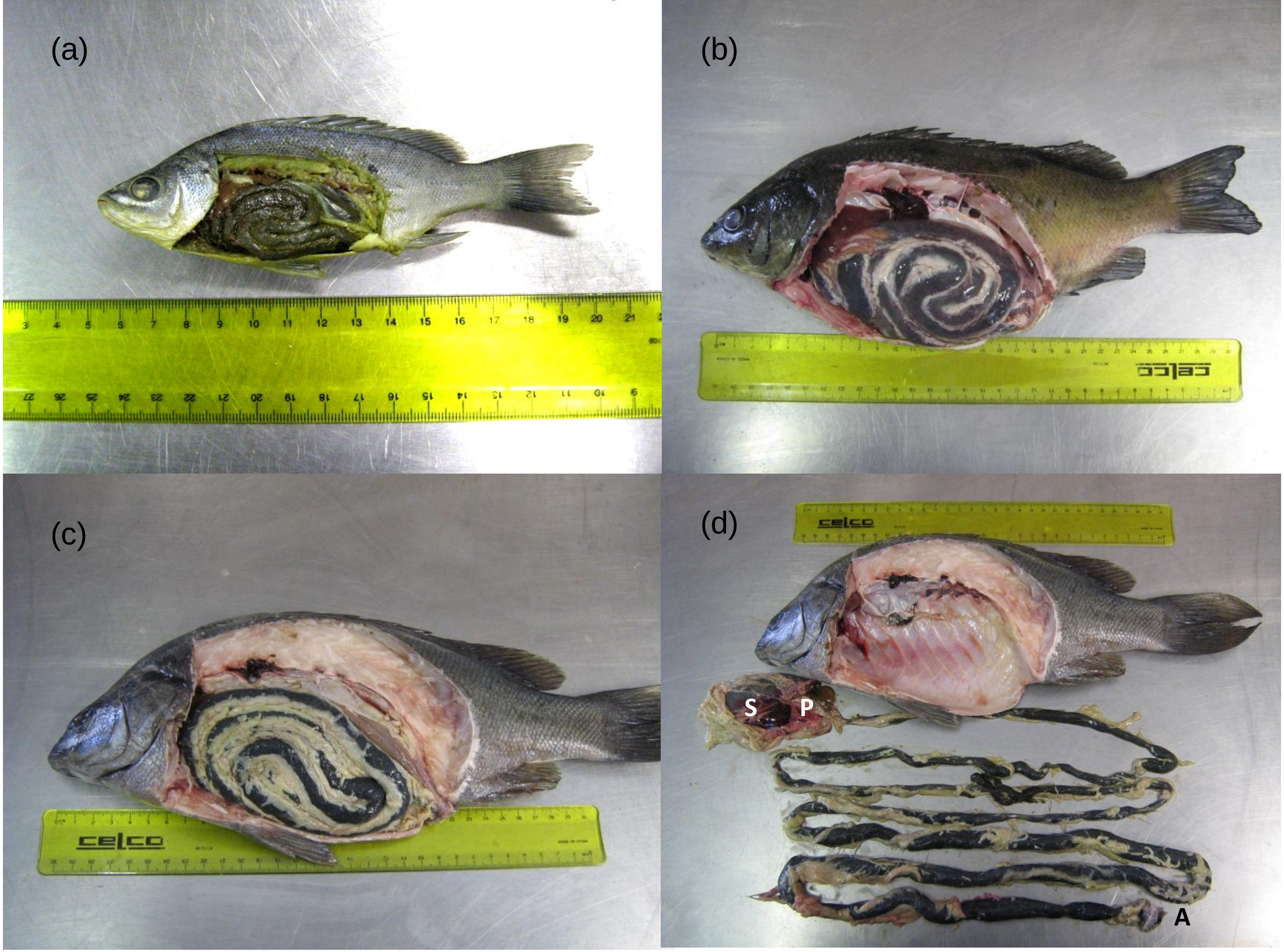

Figure S7. Terapontid intestinal morphology. (a) *Scortum parviceps* sub-adult, intestine in situ; (b) *Scortum parviceps* adult, "*Scortum*" intestine in situ (c) *Scortum parviceps* adult, "*Scortum*" intestine fully developed in situ; (d) *Scortum parviceps* adult intestine extended. S, stomach; P, pylorus; A, anus.

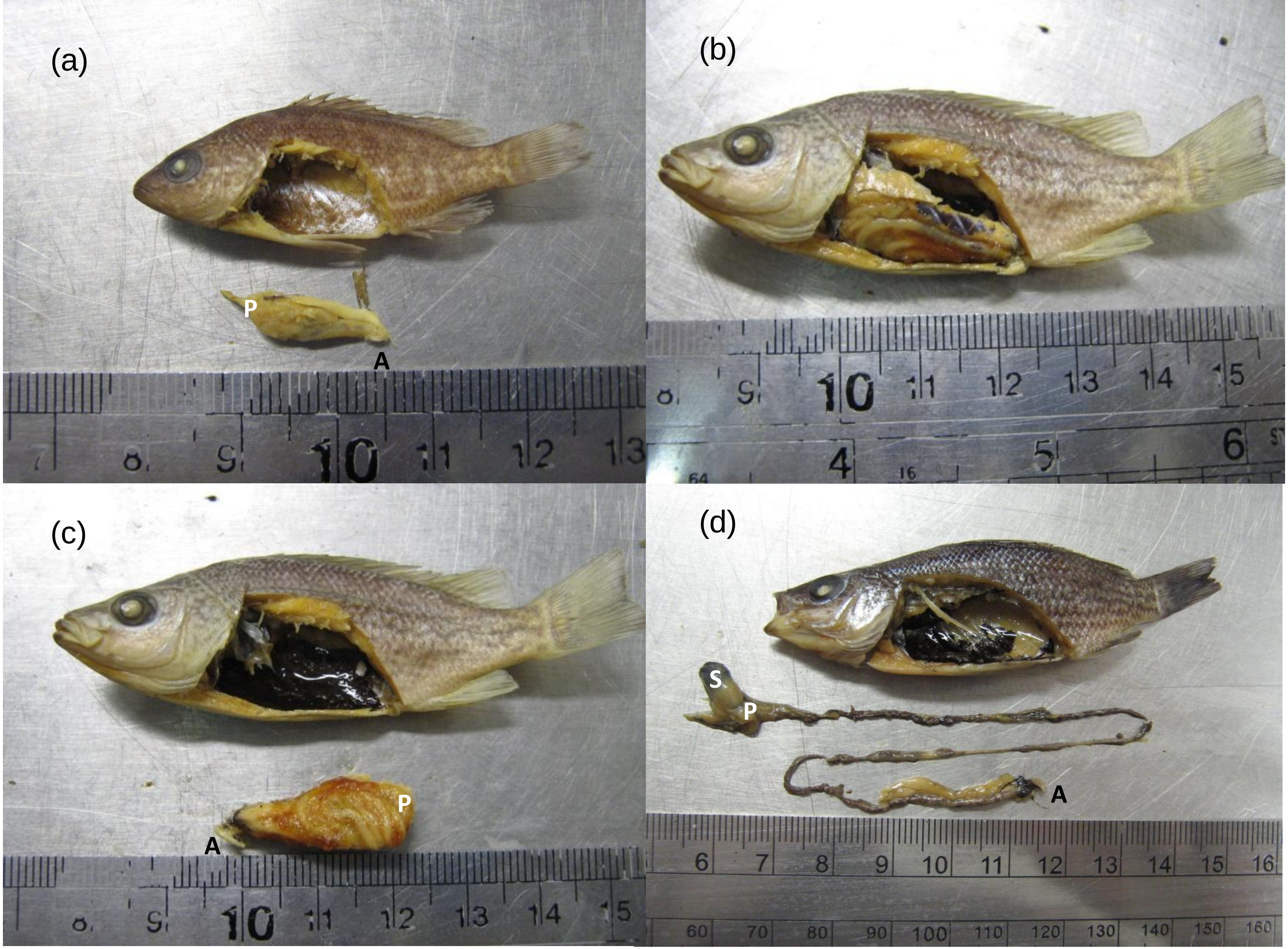

Figure S8. Terapontid intestinal morphology. (a) *Leiopotherapon aheneus* juvenile, “two-loop” intestine excised; (b) *Leiopotherapon aheneus* adult, “*L. aheneus*” intestine in situ (c) *Leiopotherapon aheneus* adult, “*L. aheneus*” intestine, excised and rotated; (d) *Leiopotherapon aheneus* adult intestine extended. S, stomach; P, pylorus; A, anus.

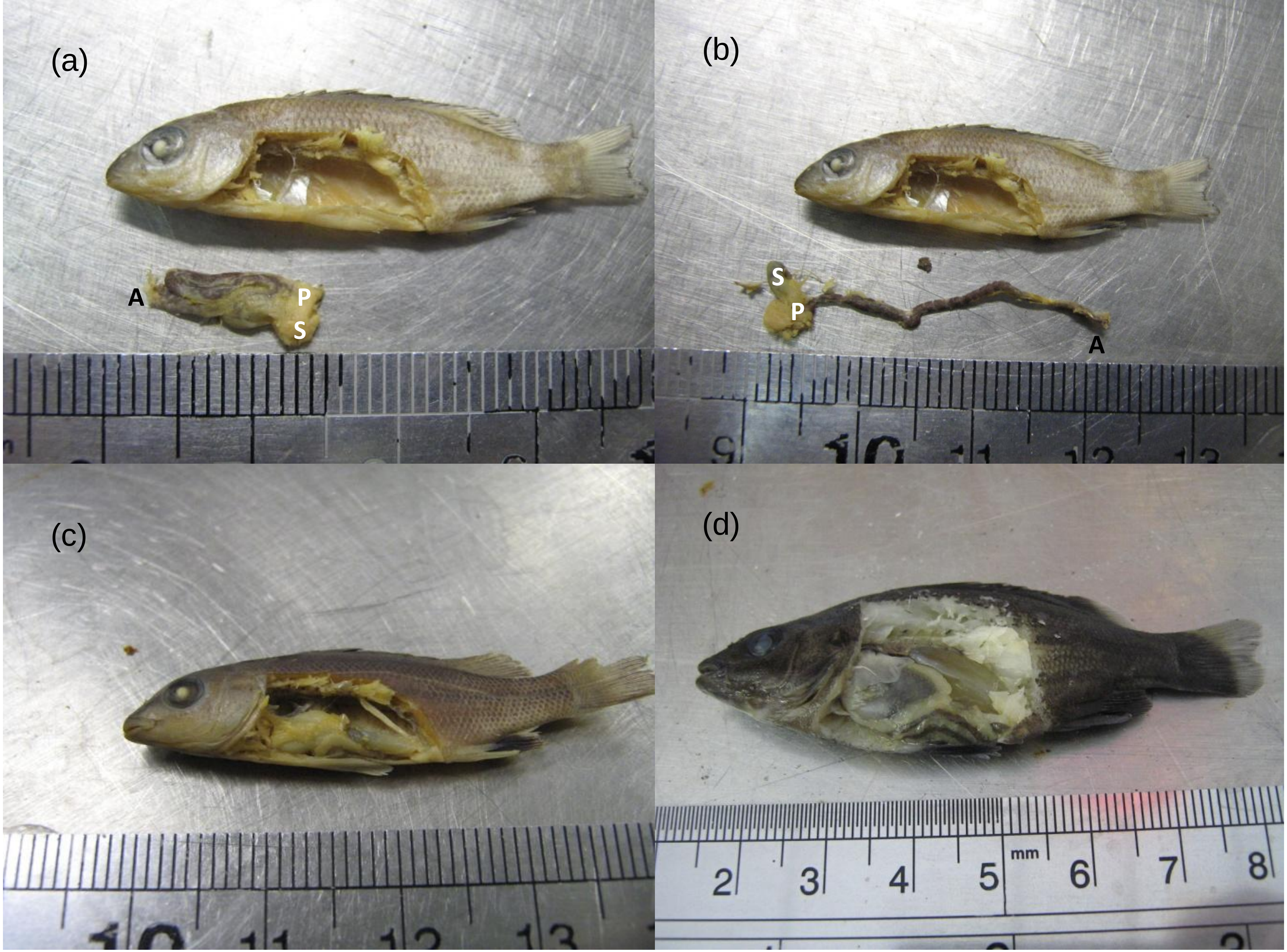

Figure S9. Terapontid intestinal morphology. (a) *Syncomistes butleri* juvenile, “two-loop” intestine, excised and rotated; (b) *Syncomistes butleri* juvenile, “two-loop” intestine fully extended; (c) *Syncomistes butleri* juvenile, early “six-loop” intestine in situ; (d) *Syncomistes butleri* juvenile, “six-loop” intestine in situ. S, stomach; P, pylorus; A, anus.

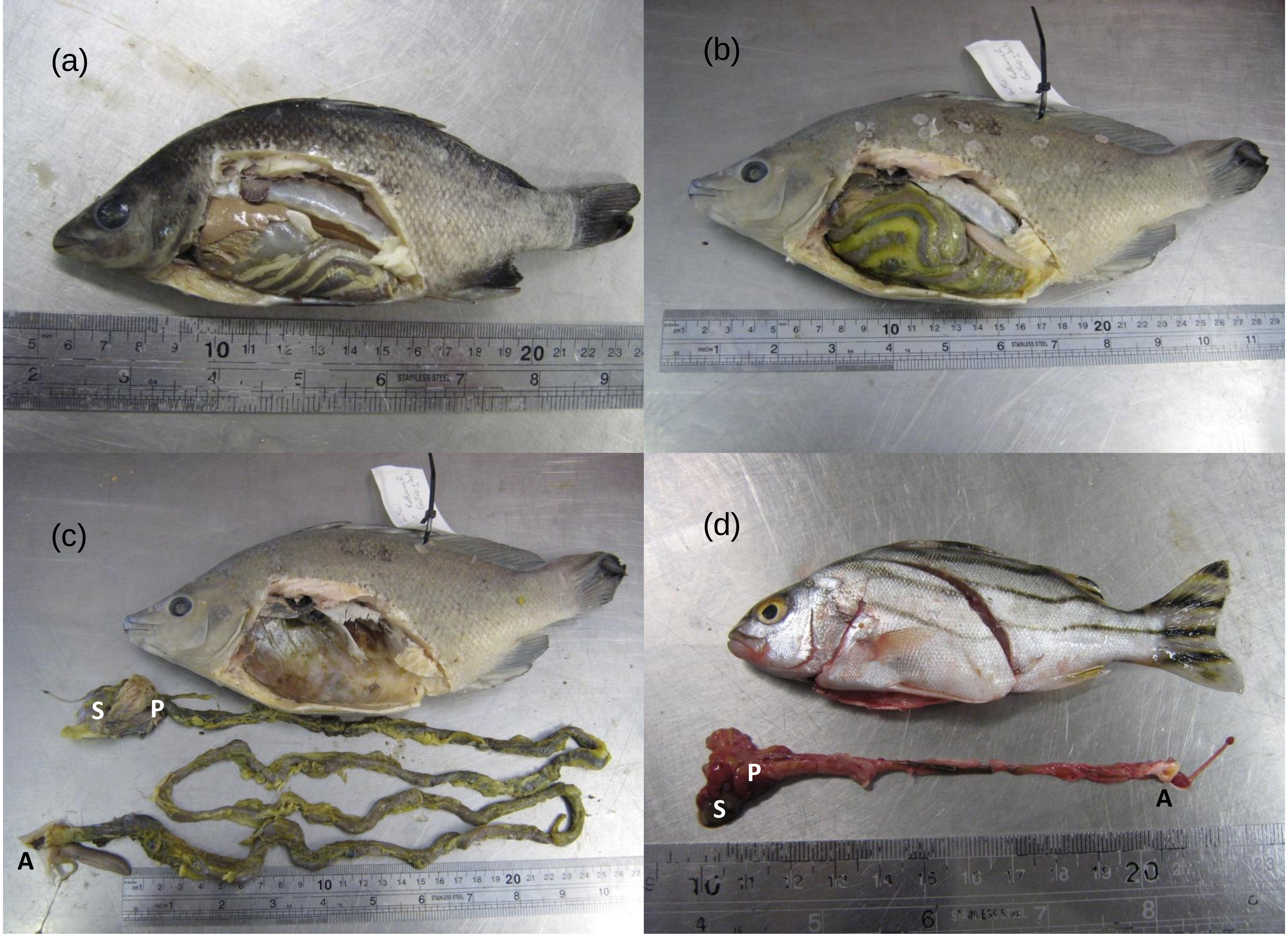

Figure S10. Terapontid intestinal morphology. (a) *Syncomistes butleri sub-adult*, intestine in situ; (b) *Syncomistes butleri* adult, "*Syncomistes*" intestine in situ; (c) *Syncomistes butleri* adult, "*Syncomistes*" intestine fully extended; (d) *Terapon jarbua* adult, "two-loop" intestine fully extended. S, stomach; P, pylorus; A, anus.

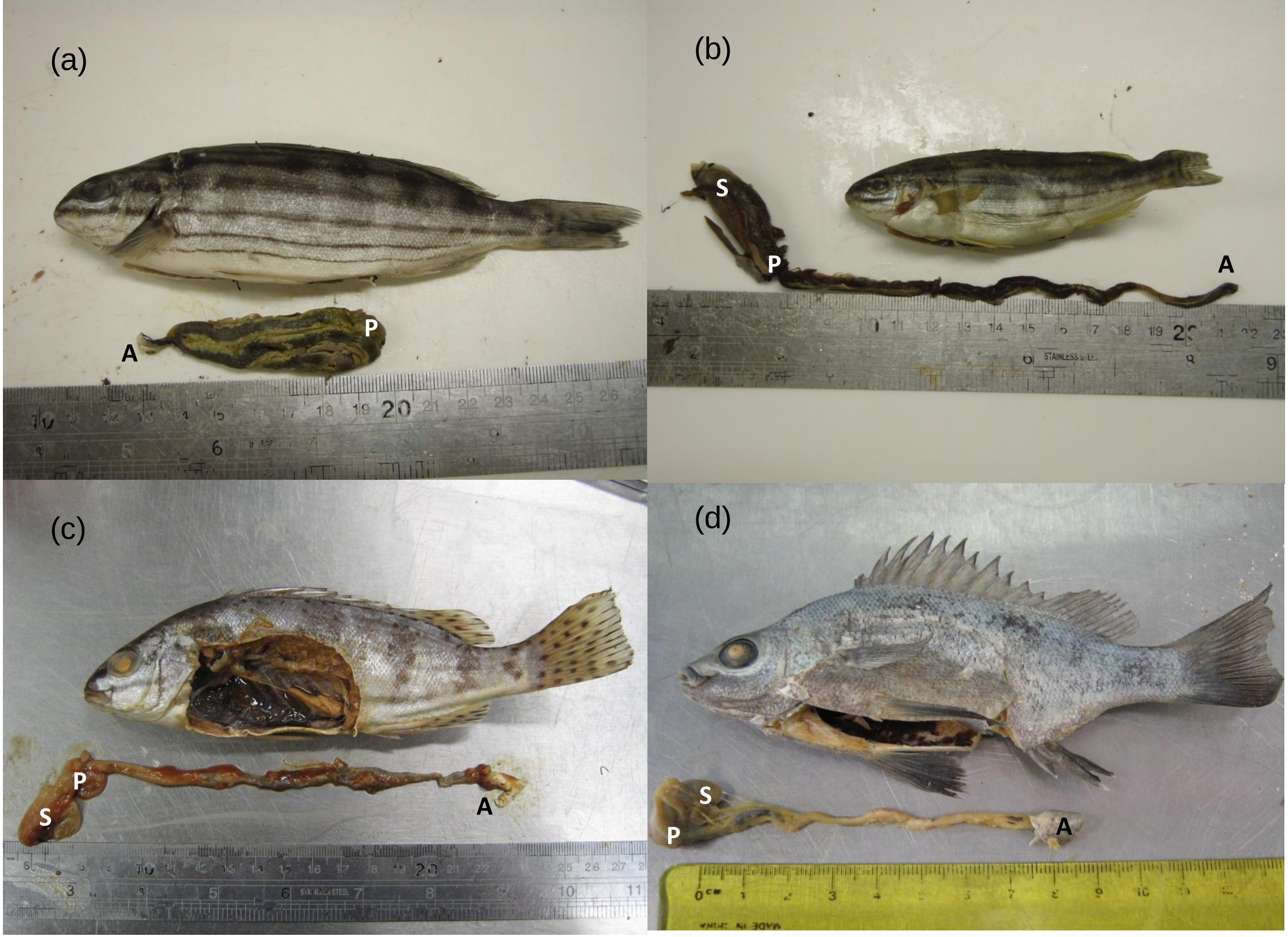

Figure S11. Terapontid intestinal morphology. (a) *Helotes sexlineatus* adult, "*Helotes*" intestine, excised and rotated; (b) *Helotes sexlineatus* adult, "*Helotes*" intestine fully extended; (c) *Pelsartia humeralis* adult, "two-loop" intestine fully extended; (d) *Variichthys lacustris* adult, "two-loop" intestine fully extended. S, stomach; P, pylorus; A, anus.

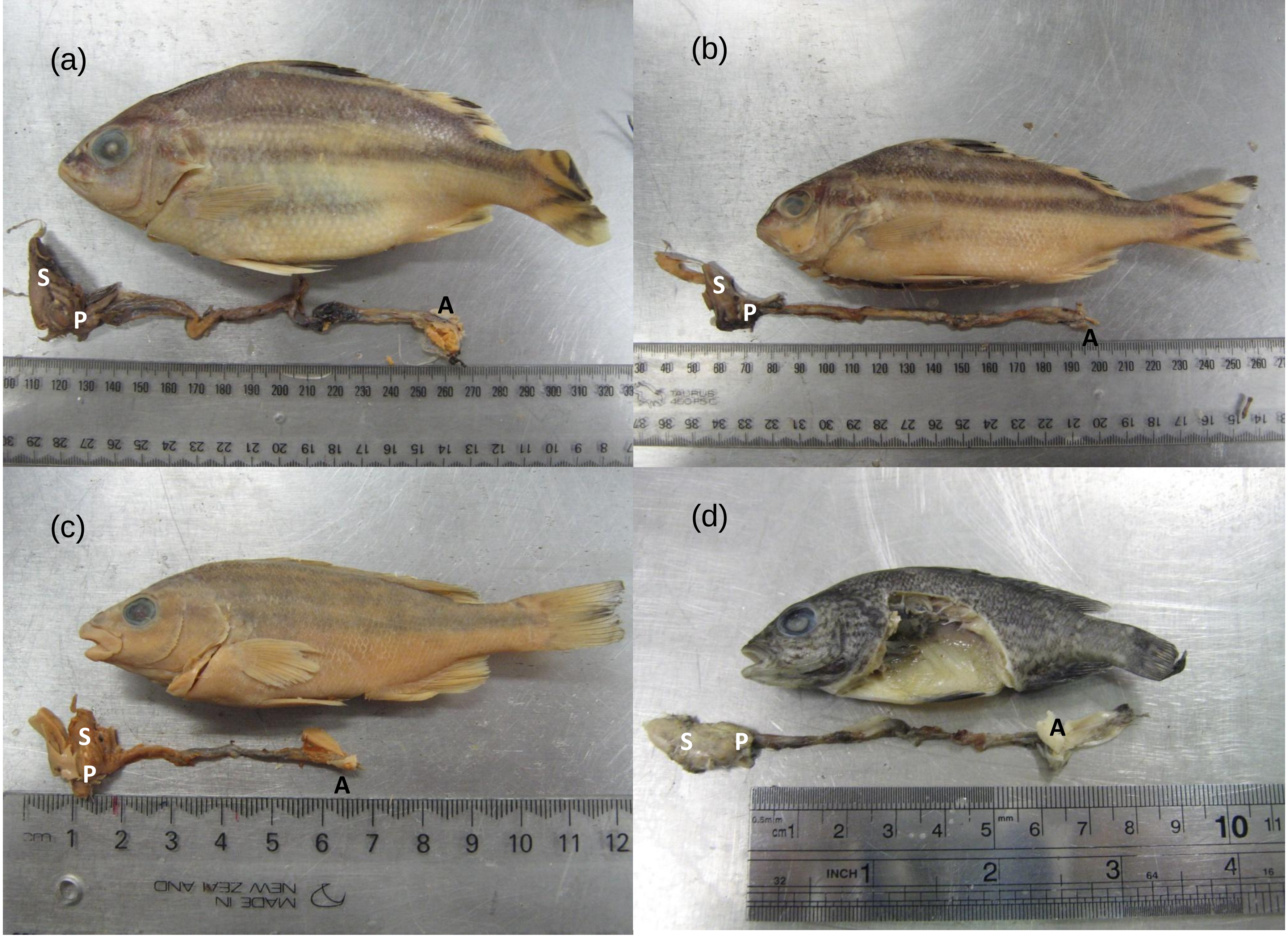

Figure S12. Terapontid intestinal morphology. (a) *Terapon theraps* adult, “two-loop” intestine fully extended; (b) *Terapon puta* adult, “two-loop” intestine fully extended; (c) *Hephaestus transmontanus* adult, “two-loop” intestine fully extended; (d) *Hephaestus carbo* adult, “two-loop” intestine fully extended. S, stomach; P, pylorus; A, anus.
