# Supplementary material for: Ontogenetic development of intestinal length and relationships to diet in an Australasian fish family (Terapontidae)
Source: BMC Evol Biol. 2013 Feb 25;13:53. doi: 10.1186/1471-2148-13-53 (PMC3598832; doi:10.1186/1471-2148-13-53)
Supplement: Additional file 3: Table S1 — Terapontid intestinal length scaling analyses. Results for scaling analyses of reduced major axis regressions of Log10 –transformed standard length versus Log10 – transformed intestinal length for 27 terapontid species. Statistically significant allometric scaling relationship (i.e., where the 95% confidence interval for slope does not overlap with an isometric slope of 1.0) are highlighted in bold. n signifies the number of intestinal length measurements per species. [file 1471-2148-13-53-S3.docx]

**Table S1.** Results for scaling analyses of reduced major axis regressions of Log_10_ –transformed standard length versus Log_10_ –transformed intestinal length for 27 terapontid species. Statistically significant allometric scaling relationship (i.e., where the 95% confidence interval for slope does not overlap with an isometric slope of 1.0) are highlighted in bold. n signifies the number of intestinal length measurements per species.

| **Species** | **Intestinal length category** | **Slope (a)** | **a (confidence limits)** | **Y-intercept** | **r²** | **n** | **Size range (mm SL)** |
| --- | --- | --- | --- | --- | --- | --- | --- |
| *Amniataba caudovittatus* | "Two-loop" | 1.299 | 0.946-1.439 | -0.635 | 0.964 | 11 | 67-163 |
| *Amniataba percoides* | "Two-loop" | **1.385** | 1.339-1.431 | -0.761 | 0.929 | 432 | 15-126 |
| *Hannia greenwayi* | "Two-loop" | **1.194** | 1.041-1.347 | -0.423 | 0.954 | 15 | 16-124 |
| *Hephaestus carbo* | "Two-loop" | **1.274** | 1.216-1.333 | -0.581 | 0.954 | 87 | 37-160 |
| *Hephaestus epirrhinos* | "Two-loop" | **1.516** | 1.26-1.772 | -1.085 | 1.000 | 3 | 193-275 |
| *Hephaestus transmontanus* | "Two-loop" | 1.767 | 0.838-5.480 | -1.619 | 0.112 | 20 | 68-84 |
| *Leiopotherapon unicolor* | "Two-loop" | **1.252** | 1.223-1.280 | -0.572 | 0.936 | 479 | 11-170 |
| *Mesopristes argenteus* | "Two-loop" | **1.257** | 1.177-1.346 | -1.183 | 0.952 | 13 | 86-223 |
| *Pelates quadrilineatus* | "Two-loop" | 0.999 | 0.602-1.270 | -0.022 | 0.891 | 7 | 93-137 |
| *Pelates sexlineatus* | "Two-loop" | **1.317** | 1.190-1.455 | -0.675 | 0.952 | 16 | 70-117 |
| *Terapon jarbua* | "Two-loop" | 0.943 | 0.818-1.02 | 0.129 | 0.894 | 32 | 39-156 |
| *Terapon puta* | "Two-loop" | 1.095 | 0.611-1.300 | -0.221 | 0.971 | 6 | 79-167 |
| *Terapon theraps* | "Two-loop" | 1.496 | 0.706-1.835 | -1.092 | 0.822 | 8 | 120-170 |
| *Varrichthys lacustris* | "Two-loop" | 1.150 | 0.427-1.246 | -0.358 | 0.941 | 12 | 39-181 |
| *Bidyanus welchi* | "Six-loop" | **1.594** | 1.282-4.464 | -1.146 | 0.769 | 8 | 131-235 |
| *Hephaestus fuliginosus* | "Six-loop" | **1.440** | 1.408-1.471 | -0.769 | 0.965 | 292 | 24-320 |
| *Hephaestus jenkinsi* | "Six-loop" | **1.574** | 1.478-1.671 | -0.988 | 0.926 | 80 | 32-280 |
| *Hephaestus tulliensis* | "Six-loop" | **1.766** | 1.291-2.241 | -1.295 | 0.780 | 16 | 81-217 |
| *Pingalla gilberti* | *"Pingalla"* | **1.470** | 1.146-1.794 | -0.625 | 0.676 | 30 | 34-97 |
| *Pingalla lorentzi* | *"Pingalla"* | **1.316** | 1.042-1.591 | -0.324 | 0.913 | 12 | 48-116 |
| *Scortum ogilbyi* | *"Scortum"* | **1.626** | 1.501-1.752 | -0.817 | 0.884 | 80 | 53-324 |
| *Scortum parviceps* | *"Scortum"* | **1.681** | 1.599-1.763 | -0.931 | 0.976 | 74 | 15-297 |
| *Syncomistes butleri* | *"Syncomistes"* | **1.820** | 1.694-1.987 | -1.214 | 0.956 | 99 | 38-241 |
| *Syncomistes rastellus* | *"Syncomistes"* | **1.938** | 1.249-2.628 | -1.373 | 0.777 | 13 | 68-166 |
| *Syncomistes trigonicus* | *"Syncomistes"* | **2.356** | 2.155-2.557 | -2.042 | 0.948 | 32 | 32-105 |
| *Helotes sexlineatus* | *"Helotes"* | **1.289** | 1.174-1.387 | -0.441 | 0.934 | 32 | 87-159 |
| *Leiopotherapon aheneus* | *"L. aheneus"* | **1.806** | 1.650-1.962 | -1.123 | 0.943 | 34 | 13-73 |
